# An antibody-PROTAC conjugate targets BRD4/c-Myc/PD-L1 to enhance immunotherapy efficacy in triple-negative breast cancer

**DOI:** 10.1016/j.xcrm.2026.102948

**Published:** 2026-07-29

**Authors:** Yunru Gu, Chen Li, Yuhan Zhao, Tianrui Xu, Ye Zhu, Dandan Wang, Xiaoning Wang, Jiaxuan Chen, Jinbo Li, Xiaoxiang Guan

**Affiliations:** 1 Department of Oncology, The First Affiliated Hospital with Nanjing Medical University, Nanjing 210029, China; 2 State Key Laboratory of Analytical Chemistry for Life Sciences, Jiangsu Key Laboratory of Advanced Organic Materials, School of Chemistry and Chemical Engineering, Chemistry and Biomedicine Innovation Center (ChemBIC), Nanjing University, Nanjing 210023, China; 3 Department of Breast Surgery, The First Affiliated Hospital with Nanjing Medical University, 300 Guangzhou Road, Nanjing 210029, China; 4 Jiangsu Key Lab of Cancer Biomarkers, Prevention and Treatment, Collaborative Innovation Center for Cancer Personalized Medicine, Nanjing Medical University, Nanjing 211166, China

**Keywords:** antibody-PROTAC conjugate, TROP2, hypoxia responsive, CD8^+^ T cell, immunotherapy, long-term immune memory, TNBC

## Abstract

Triple-negative breast cancer (TNBC) remains an aggressive malignancy lacking effective and safe treatments. Proteolysis-targeting chimeras (PROTACs) represent a promising modality for intractable diseases, but their application in TNBC is hindered by inefficient delivery. Here, we develop ASA, a TNBC-targeting antibody-PROTAC conjugate that links a TROP2-specific antibody to a BRD4-degrading PROTAC, with specificity conferred by TROP2-dependent endocytosis and hypoxia-triggered linker cleavage. ASA exhibits superior TNBC targeting and antitumor efficacy compared with its unconjugated PROTAC moiety. ASA monotherapy elicits stronger long-term immunological memory than the antibody-drug conjugate (ADC) sacituzumab govitecan, an effect that is further amplified by anti-PD-L1 combination. Mechanistically, ASA induces sustained BRD4 degradation, suppressing c-Myc and PD-L1 to inhibit tumor progression and drive long-lasting immunity. By disrupting DNA repair pathways, ASA synergizes with anti-PD-L1 to enhance tumor control and increase CD8^+^ T cell infiltration and activation. These findings position ASA as a promising strategy for safe, effective, and durable TNBC therapy.

## Highlights

• Development of a TNBC-targeting antibody-PROTAC conjugate (ASA)
• ASA exhibits enhanced targeting and efficacy compared with PROTAC alone
• ASA plus αPD-L1 improves tumor control and CD8^+^ T cell responses

## Graphical abstract

**Figure:**
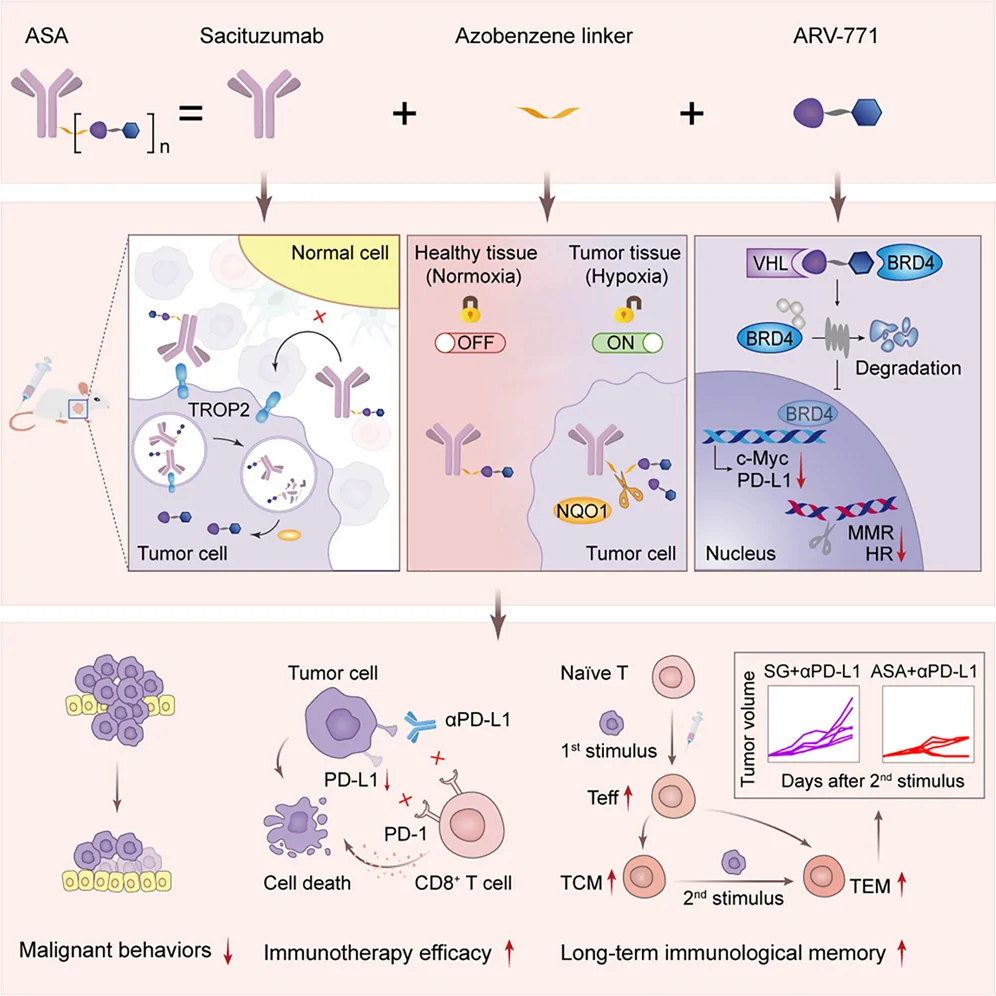

## Teaser

Gu et al. develop ASA, an antibody-PROTAC conjugate for TNBC, that integrates tumor homing, hypoxia-responsive cleavage, and PROTAC-mediated degradation. This design enables targeted degradation within tumors and promotes robust antitumor immunity and long-term immune memory when combined with αPD-L1.

## Introduction

Triple-negative breast cancer (TNBC) is the most aggressive breast cancer subtype with a poor long-term prognosis.^1^ Due to its absence of estrogen receptor (ER), progesterone receptor (PR), and human epidermal growth factor receptor 2, which are key targets for conventional breast cancer therapies, TNBC has long faced a targeted treatment dilemma.^2^ In recent years, modalities including immune checkpoint inhibitors (ICIs), antibody-drug conjugates (ADCs), and inhibitors targeting oncogenic vulnerabilities have been successfully developed and granted FDA approval.^2^^,^^3^^,^^4^^,^^5^^,^^6^^,^^7^ However, their therapeutic benefits remain limited by suboptimal efficacy and safety issues in clinical settings.^8^^,^^9^^,^^10^ Therefore, further in-depth investigations into alternative therapeutic strategies are imperative for effective and safe TNBC treatments.

Proteolysis-targeting chimera (PROTAC) represents a revolutionary shift in drug design and oncotherapy, currently advancing through late-stage clinical trials.^11^^,^^12^ PROTAC drugs enable sustained and efficient targeted protein degradation (TPD) by inducing the binding of PROTAC-protein complexes to E3 ligases.^13^^,^^14^^,^^15^ The PROTAC strategy has shown promise in degrading various “undruggable” proteins in TNBC, including BET family proteins,^16^ MDM2,^17^ and c-Myc.^18^ However, effective and safe treatment requires delivering PROTAC agents to tumor cells, as off-tumor proteolysis can cause severe systemic toxicity.^19^^,^^20^ Building on this, researchers have integrated the targeted delivery design of ADCs to develop antibody-PROTAC conjugates, which utilize tumor-specific antibodies for precise delivery to cancer cells.^21^^,^^22^ Specifically, in several studies, chimeric bromodomain-containing protein 4 (BRD4) degraders have been conjugated to anti-ROR1 for solid tumors^23^ and to anti-STEAP1 for prostate cancer or to anti-CLL1 for acute myeloid leukemia.^24^^,^^25^ In this context, TPD via antibody-PROTAC conjugate has emerged as a potentially valuable oncotherapy. Nevertheless, there are currently no antibody-PROTAC agents specifically designed to target and treat TNBC, leaving a critical gap in its therapeutic options.

In this study, we report a TNBC-targeting antibody-PROTAC conjugate (hereafter named ASA) that combines a TROP2-targeting antibody with a BRD4-degrading PROTAC via a hypoxia-sensitive linker. TROP2, expressed in nearly 90% of TNBC cases, is a validated target for the development of ADCs such as sacituzumab govitecan (SG) and datopotamab deruxtecan.^26^^,^^27^^,^^28^^,^^29^ BRD4 is a key regulator of transcriptional programs involved in tumor survival, which also activates c-Myc-mediated malignancy and immunosuppressive tumor microenvironment (TME)^30^^,^^31^ and upregulates PD-L1 to induce T cell exhaustion.^31^^,^^32^^,^^33^ Hypoxia, a key hallmark of the solid TME, offers a strategic opportunity to utilize tumor-specific linker cleavage for site-selective payload release.^34^ Our findings demonstrate that ASA is selectively internalized into TNBC cells via TROP2-mediated endocytosis, followed by hypoxia-responsive linker cleavage to release the PROTAC for BRD4 degradation and suppression of downstream c-Myc and PD-L1. Furthermore, ASA monotherapy exerts robust antitumor efficacy and displays synergistic activity when combined with anti-PD-L1 antibody (αPD-L1). More importantly, the ASA plus αPD-L1 combination enhances CD8^+^ T cell activation and provokes long-term immunological memory, driving regression in tumor rechallenge models. Taken together, these results indicate that this strategy holds promise for broadening therapeutic options and enhancing immunotherapy in TNBC.

## Results

### Design and characterization of the TNBC-targeting antibody-PROTAC conjugate

**Figure 1:**
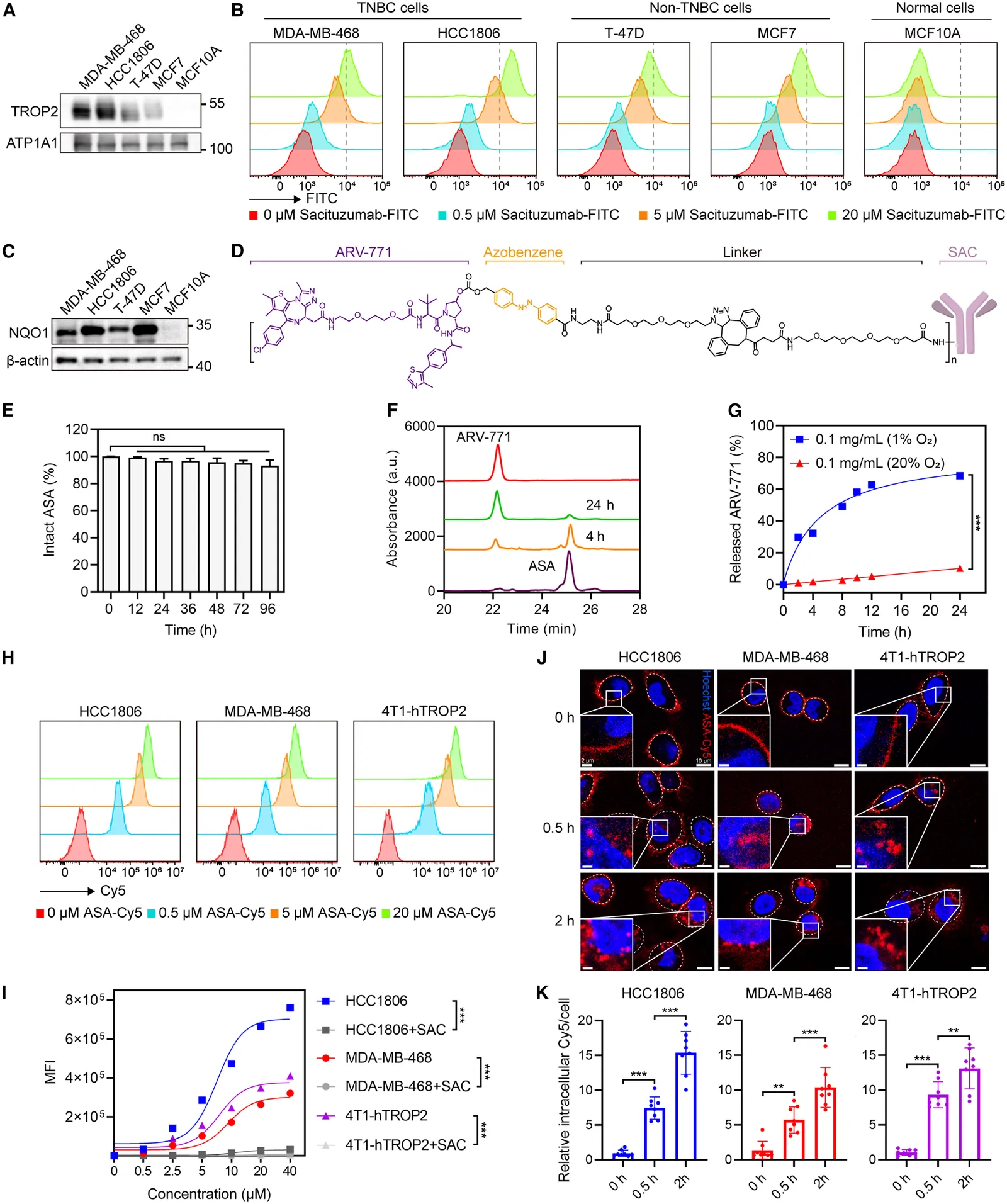
Design and characterization of the TNBC-targeting antibody-PROTAC conjugate (A) Western blot analysis of TROP2 levels in TNBC cell lines (MDA-MB-468, HCC1806), ER- and PR-positive breast cancer cell lines (T-47D and MCF7), and normal human mammary epithelial cell line (MCF10A). (B) Flow cytometry analysis of SAC binding affinity to TROP2 in different cell lines. (C) Western blot analysis of azoreductase levels in MDA-MB-468, HCC1806, T-47D, MCF7, and MCF10A cells. (D) Chemical structure of ASA. (E) High-performance liquid chromatography (HPLC) analysis showing the stability of ASA in 20% FBS at 37°C over 96 h. (F) HPLC analysis of ARV-771 release from ASA with 0.1 mg/mL azoreductase under 1% O_2_ at 4 and 24 h. (G) Quantification of HPLC analysis of ARV-771 release from ASA under 1% or 20% O_2_ at indicated time points with 0.1 mg/mL azoreductase. (H and I) Flow cytometry analysis and corresponding quantification of mean fluorescence intensity (MFI) assessing ASA binding affinity to TROP2 in TNBC cells (HCC1806, MDA-MB-468, 4T1-hTROP2) with or without 20 μM SAC pretreatment. (J and K) Cellular internalization of ASA in TNBC cells following 20-min pre-incubation at 4°C and subsequent incubation at 37°C for 0, 0.5, or 2 h. Scale bars: 10 μm and 2 μm (insets). Data are presented as mean ± SD. ns, not significant; ∗∗*p* < 0.01, ∗∗∗*p* < 0.001 by one-way ANOVA (E and K) or two-way ANOVA (G and I). See also Figures S1-S4.

Here, we designed a TNBC-targeting antibody-PROTAC conjugate, incorporating TROP2-targeting sacituzumab (SAC) and a known BRD4 PROTAC, ARV-771, as a foundation. Compared with the ER/PR-positive cell lines (T-47D and MCF7) and the normal mammary epithelial cell line (MCF10A), TNBC cell lines (MDA-MB-468 and HCC1806) showed distinct TROP2 overexpression (Figure 1A), high SAC binding (Figures 1B and S1A), and robust SAC uptake (Figure S1B), highlighting the preferential targeting potency of SAC for TNBC cells. BRD4 drives tumor progression via c-Myc/PD-L1 upregulation^31^^,^^32^ and orchestrates cancer growth and immune evasion.^30^^,^^35^ For this reason, ARV-771, a well-established PROTAC inducing near-complete BRD4 degradation across cancers,^36^^,^^37^ was selected for developing TNBC-targeting antibody-PROTAC conjugates. To conjugate SAC and ARV-771, we employed azobenzene as a linker that is cleavable by azoreductase, a tumor-specific enzyme (Figure 1C) with oxygen-regulated reductive activity, facilitating hypoxia-controlled release of azobenzene-bearing prodrugs in tumors.^38^^,^^39^

A series of TNBC-targeting antibody-PROTAC conjugates, collectively referred to as azoreductase-cleavable SAC-ARV-771 conjugate (ASA) series, were synthesized (Figure 1D). Three candidates with different drug-to-antibody ratios (DARs) were selected to evaluate BRD4 degradation in HCC1806 cells under 20% and 1% O_2_, using the same PROTAC concentration gradient of 0, 0.05, 0.1, 0.5, and 1 μM (Figures S2A and S2B). Among the tested compounds, the conjugate with a DAR of 5, which showed the strongest degradation of BRD4, was selected as the designated ASA for further examinations (unless specified otherwise, subsequent references to ASA in this study refer to this DAR = 5 variant). ASA showed favorable serum stability in 20% fetal bovine serum (FBS) and with various biological analytes, except azoreductase (Figures 1E and S3A). Under 1% O_2_ and 0.1 mg/mL azoreductase, ARV-771 release reached 32.4% at 4 h and 68.6% at 24 h (Figure 1F), with efficiency increasing at higher azoreductase concentrations (Figure S3B). In contrast, under 20% O_2_ and the same azoreductase concentration, ARV-771 release was significantly lower, at only 10.3% after 24 h (Figure 1G). These results indicate that azoreductase mediates ARV-771 release in a dose- and hypoxia-dependent manner.

Since SAC is a humanized IgG1 monoclonal antibody that targets human TROP2 (hTROP2),^40^ and the murine TNBC cell line 4T1 does not express hTROP2, we constructed a 4T1 tool cell line stably expressing hTROP2 (4T1-hTROP2) via transduction with an hTROP2-encoding lentiviral vector (Figure S4A). To assess the binding affinity of ASA for TNBC cells, we labeled ASA with Cy5 and incubated MDA-MB-468, HCC1806, and 4T1-hTROP2 cells with increasing concentrations of Cy5-labeled ASA at 4°C for 20 min. Flow cytometry analysis revealed a significant dose-dependent increase in fluorescence, indicating high binding activity of ASA toward these cells (Figure 1H). Notably, SAC pretreatment effectively blocked the binding of ASA to TNBC cells, indicating their competitive interaction for TROP2 and confirming ASA’s specific targeting of TROP2 via its antibody moiety (Figure 1I). Additionally, treatment of TNBC cells with ASA at 37°C demonstrated partial internalization by 0.5 h and majority uptake by 2 h, indicating good cellular permeability (Figures 1J and 1K). Thus, we developed a TNBC-targeting antibody-PROTAC conjugate with high binding affinity for TNBC cells and efficient intracellular delivery, rendering it promising for both cellular assays and *in vivo* applications.

### ASA effectively degrades BRD4, downregulates c-myc and PD-L1, and inhibits malignancy in TNBC cells

**Figure 2:**
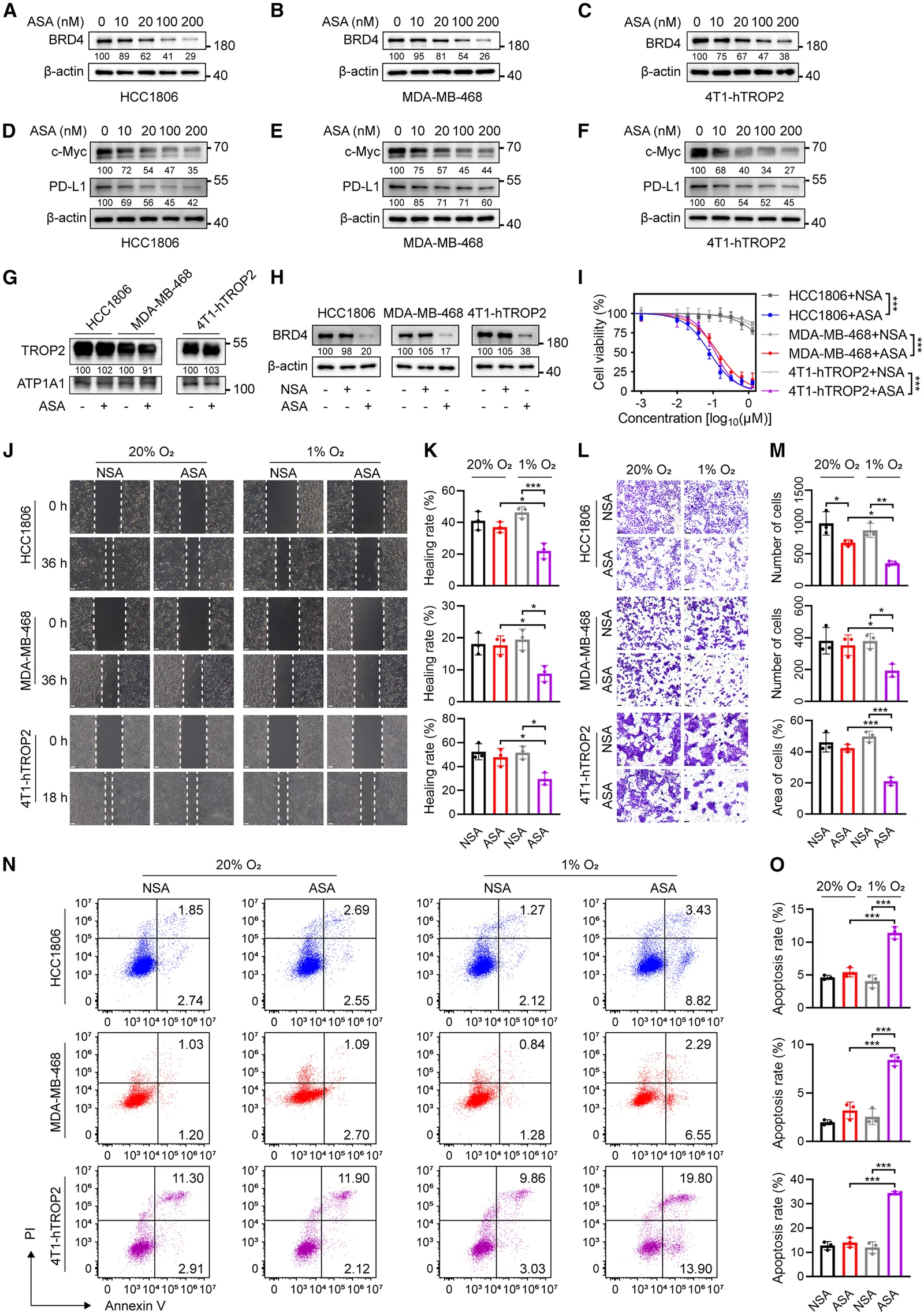
ASA effectively degrades BRD4, downregulates c-Myc and PD-L1, and inhibits malignancy in TNBC cells (A–C) Western blot analysis of BRD4 in TNBC cells (HCC1806, MDA-MB-468, and 4T1-hTROP2) treated with a concentration gradient of ASA for 6 h under 1% O_2_. (D–F) Western blot analysis of c-Myc and PD-L1 in TNBC cells treated with a concentration gradient of ASA for 24 h under 1% O_2_. (G) TROP2 expression in TNBC cells with or without 200 nM ASA treatment for 6 h under 1% O_2_. (H) Western blot analysis of BRD4 in TNBC cells treated with PBS, ASA (200 nM), or NSA (200 nM) for 6 h under 1% O_2_. (I) Cell viability of TNBC cells following 48-h treatment with a concentration gradient of ASA or NSA under 1% O_2_. (J and K) Migration of TNBC cells following treatment with ASA or NSA under 20% or 1% O_2_. Scale bars, 100 μm. (L and M) Invasion of TNBC cells following treatment with ASA or NSA under 20% or 1% O_2_. Scale bars, 100 μm. (N and O) Apoptosis of TNBC cells following 24-h treatment with ASA or NSA under 20% or 1% O_2_. Data are presented as mean ± SD. ns, not significant; ∗*p* < 0.05, ∗∗*p* < 0.01, ∗∗∗*p* < 0.001 by two-way ANOVA (I) or one-way ANOVA (K, M, and O). See also Figures S4 and S5.

To elucidate the efficacy of ASA in inducing BRD4 degradation under hypoxic conditions, TNBC cell lines (MDA-MB-468, HCC1806, and 4T1-hTROP2) were pretreated under 1% O_2_ for 24 h to simulate a hypoxic TME and then treated with ASA for 6 h under the same oxygen tension. A concentration-dependent reduction in BRD4 protein levels was observed across all tested cell lines (Figures 2A–2C; Table S1), an effect that was significantly reversed by pretreatment with the proteasome inhibitor MG132, confirming a proteasomal-dependent mechanism for ASA-mediated BRD4 degradation (Figure S4B). We further extended ASA treatment to 24 h and observed significant downregulation of c-Myc and PD-L1 (Figures 2D–2F), highlighting the pronounced capability of ASA to not only degrade BRD4 but also inhibit these downstream effectors^31^^,^^32^ in TNBC cells under hypoxic conditions. Moreover, TROP2 remained stably expressed on TNBC cells following ASA treatment (Figure 2G), supporting efficient receptor recycling to ensure continuous drug action. Notably, at equivalent PROTAC molar doses, ARV-771 exhibited superior BRD4 degradation efficacy after 6 h of treatment in TNBC cells (Figures S4C–S4E; Table S1). This is likely due to the small-molecule nature of ARV-771, which enables more rapid cellular entry and faster target engagement than ASA, whose internalization relies on antibody-mediated endocytosis. Nevertheless, the effective downregulation of c-Myc and PD-L1 at 24 h with either ASA or ARV-771 underscores that ASA achieves potent downstream inhibition despite its slower degradation kinetics (Figures 2D–2F and S4F–S4H). These findings demonstrate that ASA, by virtue of its PROTAC payload, induces specific BRD4 degradation and subsequent downregulation of c-Myc and PD-L1 across multiple TNBC cell lines under hypoxic conditions.

To clarify the specificity of azoreductase-mediated cleavage, we synthesized a non-cleavable SAC-ARV-771 conjugate (NSA) as a control. In contrast to ASA, NSA failed to degrade BRD4 in TNBC cells under 1% O_2_, indicating a dependence on azoreductase-mediated ARV-771 release (Figure 2H). Consistently, NSA exerted a negligible effect on the growth of TNBC cells, whereas ASA significantly impeded proliferation in TNBC cells (Figure 2I; Table S2). Since azoreductase is an oxygen-sensitive enzyme that cleaves the N=N bonds of azo compounds under hypoxic conditions,^38^ the activity of ASA on TNBC cell motility and apoptosis should also be hypoxia dependent in theory. Our results showed that ASA significantly inhibited TNBC cell migration and invasion under 1% O_2_ compared with 20% O_2_, while NSA had no similar effect (Figures 2J–2M). In line with these findings, flow cytometry analysis revealed a significant increase in apoptosis rate following ASA treatment under 1% O_2_, which was negligible under 20% O_2_ or with NSA treatment (Figure 2N and 2O). Mechanistically, c-Myc overexpression rescued the phenotypes induced by ASA, demonstrating an essential role of c-Myc in mediating ASA’s antitumor effects (Figures S5A–S5F). Taken together, these data suggest that ASA-induced suppression of the BRD4/c-Myc axis effectively inhibits TNBC cell proliferation, migration, and invasion, while promoting apoptosis under hypoxic conditions.

### TROP2- and hypoxia-based selectivity enables ASA to exert precise BRD4 degradation and anti-proliferative effects on TNBC cells

**Figure 3:**
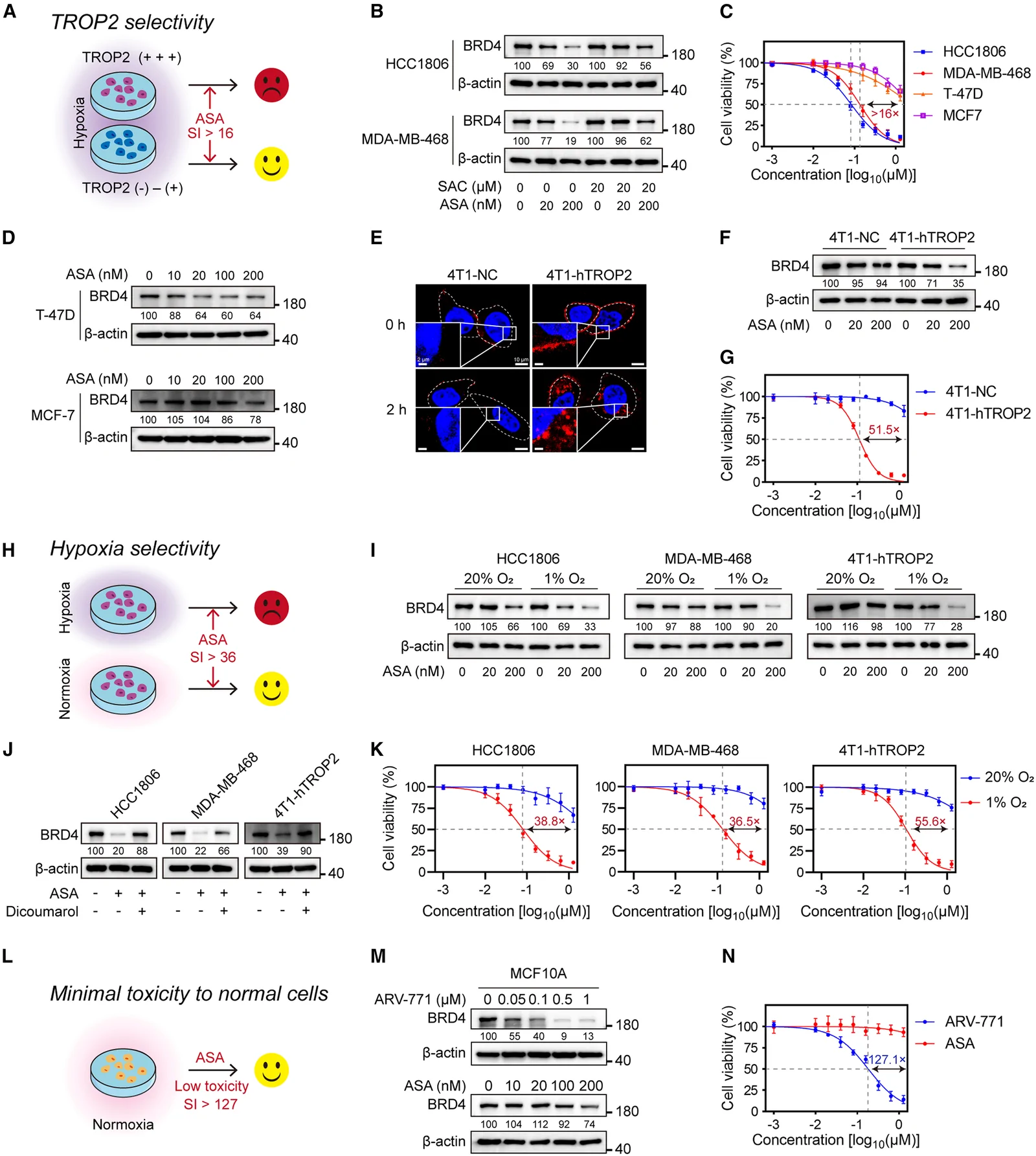
TROP2- and hypoxia-based selectivity enables ASA to exert precise BRD4 degradation and anti-proliferative effects on TNBC cells (A) Schematic illustrating TROP2-based selectivity of ASA. Selectivity index (SI) was calculated based on the IC_50_ of indicated cells. (B) Western blot analysis of BRD4 in TNBC cells (HCC1806 and MDA-MB-468) after 20-min preincubation with vehicle or SAC (20 μM) at 4°C, followed by washout and 6-h treatment with ASA in a concentration gradient under 1% O_2_. (C) Cell viability of TNBC cells and ER- and PR-positive breast cancer cells (T-47D and MCF7) following 48-h treatment with a concentration gradient of ASA under 1% O_2_. (D) Western blot analysis of BRD4 in non-TNBC cells (T-47D and MCF7) treated with a concentration gradient of ASA under 1% O_2_. (E) Cellular internalization of ASA in 4T1-NC and 4T1-hTROP2 cells following 20-min pre-incubation at 4°C and subsequent incubation at 37°C for 0 or 2 h. Scale bars: 10 μm and 2 μm (insets). (F) Western blot analysis of BRD4 in 4T1-NC and 4T1-hTROP2 cells treated with ASA at different concentrations under 1% O_2_. (G) Cell viability of 4T1-NC and 4T1-hTROP2 following 48-h treatment with a concentration gradient of ASA under 1% O_2_. (H) Schematic illustrating hypoxia-based selectivity of ASA. SI was calculated based on the IC_50_ of TNBC cells under indicated oxygen concentrations. (I) Western blot analysis of BRD4 in TNBC cells treated with a concentration gradient of ASA for 6 h under 20% or 1% O_2_. (J) Western blot analysis of BRD4 in TNBC cells pretreated with DMSO (control) or dicoumarol (10 μM) for 4 h, followed by 6-h ASA treatment under 1% O_2_. (K) Cell viability of TNBC cells following 48-h treatment with a concentration gradient of ASA under 20% or 1% O_2_. (L) Schematic illustrating minimal toxicity of ASA to normal cells. SI was calculated based on the IC_50_ of MCF10A treated with indicated agents. (M) Western blot analysis of BRD4 in MCF10A treated with a concentration gradient of ARV-771 or ASA at the same PROTAC doses for 6 h under 20% O_2_. (N) Cell viability of MCF10A following treatment with a concentration gradient of ARV-771 or ASA under 20% O_2_. Data are presented as mean ± SD.

Given that ASA is composed of TROP2-targeting SAC and BRD4 PROTAC via a hypoxia-sensitive linker, we further investigated whether ASA can precisely exhibit its effect through TROP2 and hypoxia selectivity. Our results showed that high-dose SAC pretreatment attenuated ASA-induced BRD4 degradation in HCC1806 and MDA-MB-468 cells (Figure 3B), as SAC-mediated TROP2 blockade inhibited ASA’s TROP2 binding (Figure 1I). To determine whether ASA inhibits cell proliferation in a TROP2-dependent manner, we performed CCK-8 assays and calculated the selectivity index (SI) via IC_50_ comparison across multiple breast cancer cell lines with distinct TROP2 expression (Figure 1A). Results demonstrated that ASA exerted a significant inhibitory effect on cell growth in TNBC cells (HCC 1806 and MDA-MB-468), compared with a modest effect in non-TNBC cells (T-47D and MCF7), with an SI > 16 (Figures 3A and 3C). These findings were consistent with the limited BRD4 degradation observed in non-TNBC cells following ASA treatment (Figure 3D). Moreover, ASA rapidly bound to and was internalized by 4T1-hTROP2 cells, with minimal nonspecific uptake in TROP2-negative 4T1-NC controls (Figure 3E). Accordingly, ASA exhibited more effective BRD4 degradation and greater inhibition of proliferation in 4T1-hTROP2 cells than in control cells, yielding an SI of 51.5 (Figures 3F and 3G). Additionally, ASA treatment induced pronounced BRD4 reduction in TNBC cells under 1% O_2_, but a modest effect under 20% O_2_ (Figure 3I). This degradation was abrogated by pretreatment with the azoreductase inhibitor dicoumarol (Figure 3J), confirming its dependence on both hypoxic conditions and enzymatic activity. It also more potently inhibited cell proliferation under 1% O_2_ compared with 20% O_2_, with a hypoxia SI over 36, highlighting marked hypoxia-specific activity against TNBC cells (Figures 3H and 3K), which was consistent with previous findings on TNBC cell migration, invasion, and apoptosis. On the contrary, at the same PROTAC dose as ARV-771, ASA, with its dual-targeting design, exerted a negligible effect on BRD4 protein in MCF10A cells, whereas ARV-771 induced robust BRD4 degradation (Figure 3M). In line with this finding, MCF10A cell survival was significantly reduced following ARV-771 treatment compared with ASA, with an SI exceeding 127 (Figure 3L and 3N). Overall, these results elucidate that ASA specifically targets TROP2-overexpressing TNBC cells in a hypoxia-dependent manner, while showing minimal toxicity toward normal cells.

### Biodistribution, biosafety, and antitumor efficacy of ASA monotherapy in TROP2-positive TNBC models

**Figure 4:**
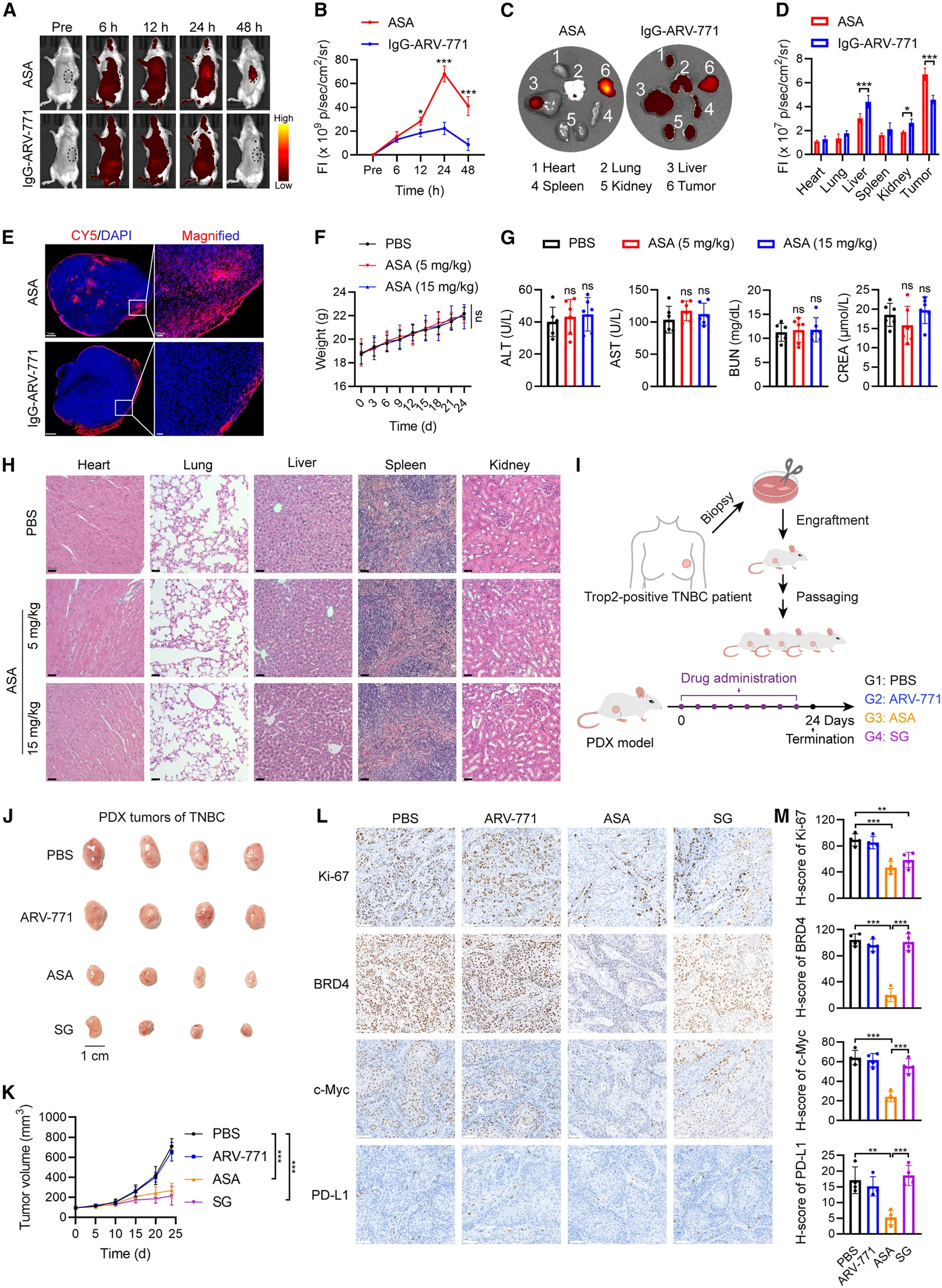
Biodistribution, biosafety, and antitumor efficacy of ASA monotherapy in TROP2-positive TNBC models (A and B) *In vivo* fluorescence imaging (A) and fluorescence intensity (FI) quantification (B) of the biodistribution of Cy5-labeled ASA and IgG-ARV-771 at indicated time points in 4T1-hTROP2-bearing mice (*n* = 4 mice per group). (C and D) Fluorescence images (C) and FI quantification (D) of Cy5-labeled ASA and IgG-ARV-771 in tissues 48 h post-administration. (E) Immunofluorescence showing the distribution of Cy5-labeled ASA and IgG-ARV-771 in tumor tissues. Scale bars: 1 mm and 100 μm (insets). (F) Body weight changes of mice treated with PBS or ASA (5 or 15 mg/kg, intravenous [i.v.], q3d) over 24 days (*n* = 6 mice per group). (G and H) Biosafety evaluation. Serum levels of alanine aminotransferase (ALT), aspartate aminotransferase (AST), blood urea nitrogen (BUN), and creatinine (CREA) (G) and representative H&E staining of major organs (heart, lung, liver, spleen, kidney) (H). Scale bars, 100 μm. (I) Schematic of patient-derived tumor xenograft (PDX) model and treatment regimens. PDX-bearing mice received i.v. injections of PBS, ARV-771 (0.32 mg/kg), ASA (10 mg/kg), or SG (10.27 mg/kg) every 3 days for 30 days (*n* = 4 mice per group). (J and K) Representative PDX tumors (J) (scale bar, 1 cm) and tumor growth curves (K). (L and M) Representative immunohistochemical (IHC) images (L) and quantification (M) of Ki-67, BRD4, c-Myc, and PD-L1 in PDX tumors from each treatment group. Data are presented as mean ± SD. ns, not significant; ∗*p* < 0.05, ∗∗*p* < 0.01, ∗∗∗*p* < 0.001 by one-way ANOVA (G and M), two-way ANOVA (B, F, and K), or unpaired Student’s *t* test (D). See also Figures S6 and S7.

Given the effective targeting capacity of ASA *in vitro*, we further explored its *in vivo* pharmacokinetic profile by intravenously administering ASA, alongside IgG-ARV-771 (a control drug developed by replacing the SAC component of ASA with IgG to abolish its TROP2-targeting activity), to mice bearing 4T1-hTROP2 tumors. Cy5-labeled ASA showed optimal tumor accumulation, with peak biodistribution at 24 h, and remained predominantly localized in tumors at 48 h, whereas IgG-ARV-771 exhibited significantly lower tumor distribution (Figures 4A and 4B). At 48 h post-injection, ASA was significantly enriched in tumors from dissected mice, whereas IgG-ARV-771 accumulated in both tumors and organs (Figures 4C and 4D). Histological analysis further confirmed that ASA was widely distributed throughout tumors, in contrast to IgG-ARV-771, which localized only at tumor peripheries (Figure 4E). Notably, ASA showed sustained tumor retention over 96 h (41.9% of peak at 72 h, 17.3% at 96 h) alongside progressive intratumoral ARV-771 accumulation from 24 to 72 h and undetectable systemic exposure (Figures S6A–S6D), confirming tumor-specific payload release and supporting a q3d (every 3 days) intravenous dosing regimen. In contrast, when free ARV-771 was administered at an equivalent PROTAC dose (0.32 mg/kg, corresponding to 10 mg/kg ASA), its intratumoral levels was barely detectable (Figures S6C). Additionally, 24-day treatment with ASA at increasing doses had no adverse effects on mouse body weight or biochemical profiles (alanine aminotransferase [ALT], aspartate aminotransferase [AST], blood urea nitrogen [BUN], and creatinine [CREA]), and histological examination confirmed no significant organ impairment (Figures 4F–4H). These results collectively validate that ASA is a safe agent with robust *in vivo* TNBC-targeting efficacy.

Based on the favorable *in vitro* activity, biodistribution, and safety profile, we next evaluated the therapeutic potential of ASA in NCG mice using a patient-derived tumor xenograft (PDX) established from a treatment-naive, TROP2-positive TNBC patient (Figures 4I, S7A, and S7B). For comparison, the unconjugated PROTAC ARV-771 was administered at an equivalent PROTAC molar dose (0.32 mg/kg, matching the PROTAC content of 10 mg/kg ASA), alongside the clinically approved TROP2-directed ADC SG (10.27 mg/kg, providing an equimolar SAC dose to ASA) (Figure 4I). Remarkably, ASA treatment achieved a tumor growth inhibition (TGI) rate of 62.2%, significantly outperforming ARV-771 (7.7%) and showing comparable efficacy to SG (69.9%) (Figures 4J and 4K). Moreover, ASA induced on-target BRD4 degradation and downregulation of c-Myc and PD-L1 in tumor tissues (Figure 4L and 4M), aligning with its mechanism and underscoring its potential to enhance antitumor immunity. These data establish the therapeutic relevance of ASA monotherapy in a clinically representative model, supporting its potential for TNBC treatment.

### ASA inhibits DNA repair pathways and induces pronounced tumor suppression in combination with αPD-L1

**Figure 5:**
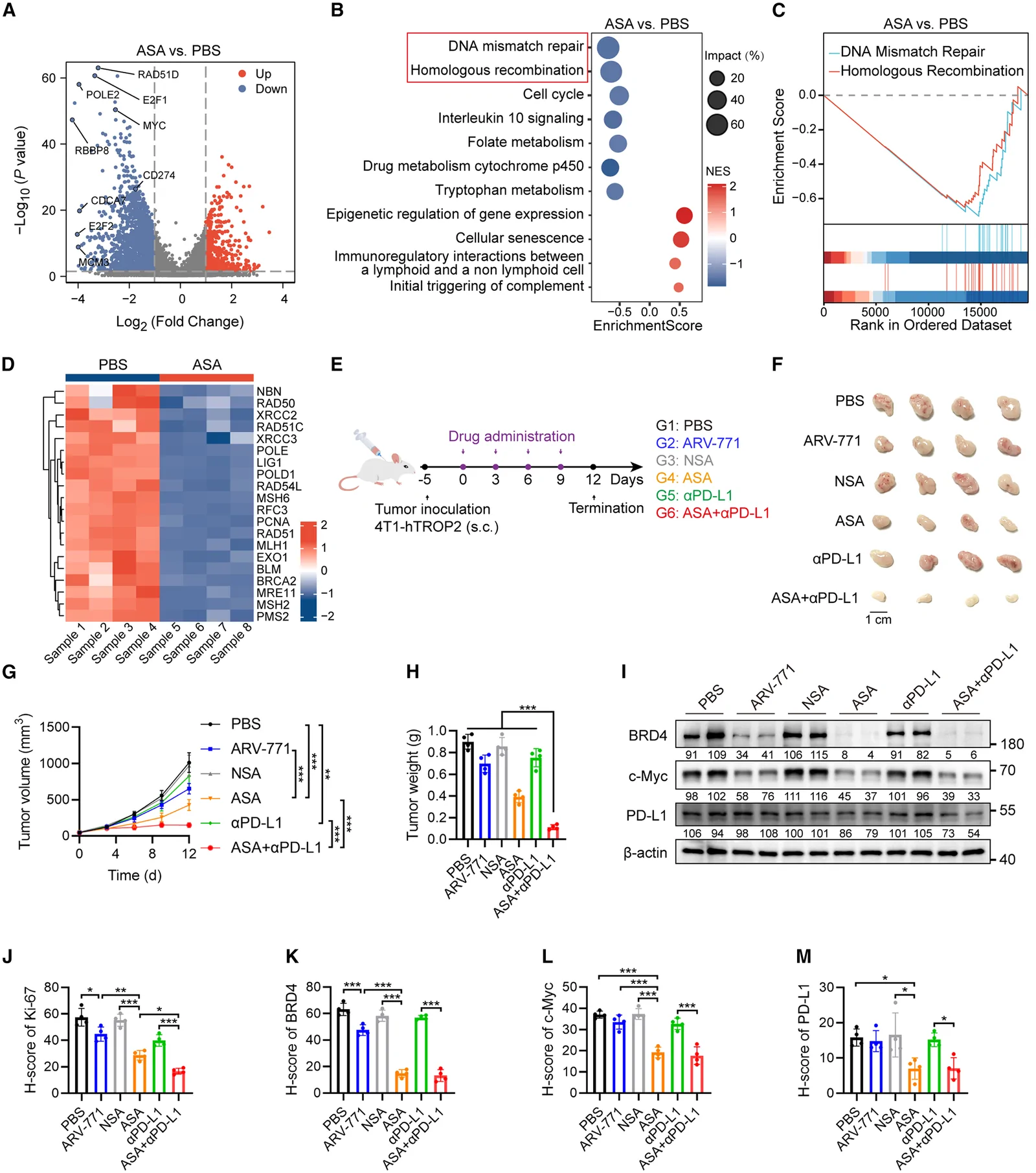
ASA inhibits DNA repair pathways and induces pronounced tumor suppression in combination with αPD-L1 (A) Volcano plot showing differentially expressed genes (ASA vs. PBS; |log2FC|>1, *p* < 0.05) in HCC1806 cells after 24 h of treatment. (B and C) GSEA analysis of the top enriched pathways (B) and the enrichment of DNA mismatch repair (MMR) and homologous recombination (HR) pathways (C) in ASA-treated vs. PBS-treated cells. (D) Heatmap of MMR/HR-related gene expression in each sample. (E) Schematic illustrating the treatment regimens. 4T1-hTROP2-bearing mice were treated for 12 days with PBS (i.v., q3d), ARV-771 (5 mg/kg, i.v., q3d), NSA (10 mg/kg, i.v., q3d), ASA (10 mg/kg, i.v., q3d), αPD-L1 (5 mg/kg, intraperitoneal [i.p.], q3d), or the combination of ASA (10 mg/kg, i.v., q3d) plus αPD-L1 (5 mg/kg, i.p., q3d) (*n* = 4 mice per group). (F–H) Tumor image (scale bar, 1 cm) (F), tumor growth curves (G), and tumor weights (H). (I) Western blot analysis of BRD4, c-Myc, and PD-L1 in 4T1-hTROP2 tumor tissues from each treatment group. (J–M) Quantification of Ki-67, BRD4, c-Myc, and PD-L1 from IHC staining of 4T1-hTROP2 tumor tissues from each treatment group. Data are presented as mean ± SD. ns, not significant; ∗*p* < 0.05, ∗∗*p* < 0.01, ∗∗∗*p* < 0.001 by two-way ANOVA (G) or one-way ANOVA (H and J–M). See also Figures S8–S12.

Although ASA functions as a BRD4 degrader to downregulate c-Myc and PD-L1, its broader biological effects in TNBC cells remain unclear, particularly given BRD4’s role as a crucial transcriptional coactivator. To address this, we performed RNA sequencing transcriptome analysis on HCC1806 cells treated with ASA or PBS. The results showed that ASA induced reprogramming of the transcriptional network, significantly downregulating cell cycle genes, such as E2F family members, POLE2, CDCA7, and MCM3, and DNA repair genes, such as RAD51D and RBBP8, compared with control (Figure 5A). Gene set enrichment analysis (GSEA) revealed that ASA significantly downregulated DNA mismatch repair (MMR) and homologous recombination (HR) pathways in TNBC cells (Figures 5B and 5C), with individual samples showing decreased expression of crucial MMR/HR genes, including MSH2, MSH6, MLH1, PMS2, BRCA2, and RAD51 (Figure 5D). Notably, MSH2, MLH1, BRCA1, and RAD51 were markedly downregulated in TNBC cells following BRD4 knockdown (Figures S8A and S8B), suggesting a direct link between BRD4 degradation and impaired DNA repair. Functionally, ASA treatment significantly delayed γH2AX clearance following camptothecin-induced DNA damage (Figures S8C and S8D), indicating a pronounced DNA repair deficiency compared with PBS controls, an effect that was reversed in c-Myc-overexpressing cells (Figures S8E and S8F), corroborating the involvement of the BRD4-c-Myc axis in mediating ASA’s activity.

MMR and HR are evolutionarily conserved processes ensuring genomic integrity, and their deficiencies can elevate tumor mutation burden (TMB).^41^^,^^42^ High TMB generates mutant peptides that are processed and recognized as neoantigens by the immune system, ultimately inducing immune cell infiltration and conferring a favorable response to ICIs.^43^^,^^44^ As a result, the established efficacy of ASA in targeting BRD4/c-Myc/PD-L1 and DNA repair pathways, together with our previously validated combined therapy strategies,^18^^,^^45^^,^^46^ supports the rationale for combining it with αPD-L1 in our therapeutic regimen to synergistically enhance immune responsiveness.

The *in vivo* therapeutic efficacy of ASA, along with its synergistic effect in combination with αPD-L1, was evaluated in immunocompetent mice following the experimental design outlined in Figure 5E. At the end of the experimental period, 5 mg/kg ARV-771 and 10 mg/kg ASA inhibited tumor growth by 35.5% and 57.8%, respectively, whereas 10 mg/kg NSA had no inhibitory effect (Figures 5F–5H). Monotherapy with 5 mg/kg αPD-L1 moderately suppressed tumor growth by 18.2%, which was significantly enhanced when combined with ASA, resulting in a TGI rate of 85.4% (Figures 5F–5H). Furthermore, the effects of ASA on BRD4 degradation and c-Myc inhibition in 4T1-hTROP2 tumors were consistent with *in vitro* findings and superior to those of ARV-771 (Figure 5I). However, its suppression of PD-L1 was less pronounced, supporting the rationale for the strategy involving αPD-L1 to block residual PD-L1. As also evidenced by histological quantification, the antitumor effects of ASA monotherapy and combination regimens were accompanied by reduced Ki-67 levels and downregulated BRD4, c-Myc, and PD-L1 expression in tumor tissues (Figures 5J–5M and S9). Importantly, these regimens were well tolerated, as there was no obvious weight loss, no abnormalities in critical markers (ALT, AST, BUN, and CREA), and no evidence of physiological impairment (Figures S10A–S10C). However, BRD4 expression analysis in liver and kidney revealed that ARV-771 significantly degraded BRD4 in healthy tissues, suggesting potential adverse events with prolonged administration, whereas ASA had no such effects (Figures S10D and S10E). Taken together, these findings demonstrate that the combination of ASA and αPD-L1 exerts synergistic effects to inhibit TNBC growth and possesses a favorable safety profile, positioning it as a promising therapeutic strategy.

To specifically verify whether the antitumor efficacy of ASA depends on its *in vivo* targeting capacity, we treated 4T1-hTROP2 tumor-bearing mice with 0.32 mg/kg ARV-771 (equimolar to the PROTAC dose in 10 mg/kg ASA) and, separately, treated 4T1-NC tumor-bearing mice with 10 mg/kg ASA (Figures S11A and S12A). Results showed that 0.32 mg/kg ARV-771 failed to exert significant tumor inhibition in the 4T1-hTROP2 model and 10 mg/kg ASA also showed no significant effect in the 4T1-NC model (Figures S11B–S11D and S12B–S12D), with both models exhibiting negligible BRD4 degradation and Ki-67 reduction (Figures S11E, S11F, S12E, and S12F). Thus, the antitumor activity of this TNBC-targeting antibody-PROTAC drug relies critically on its targeting specificity.

### ASA and SG synergize with αPD-L1 to comparably suppress metastasis and potentiate CD8^+^ T cell infiltration and activation

**Figure 6:**
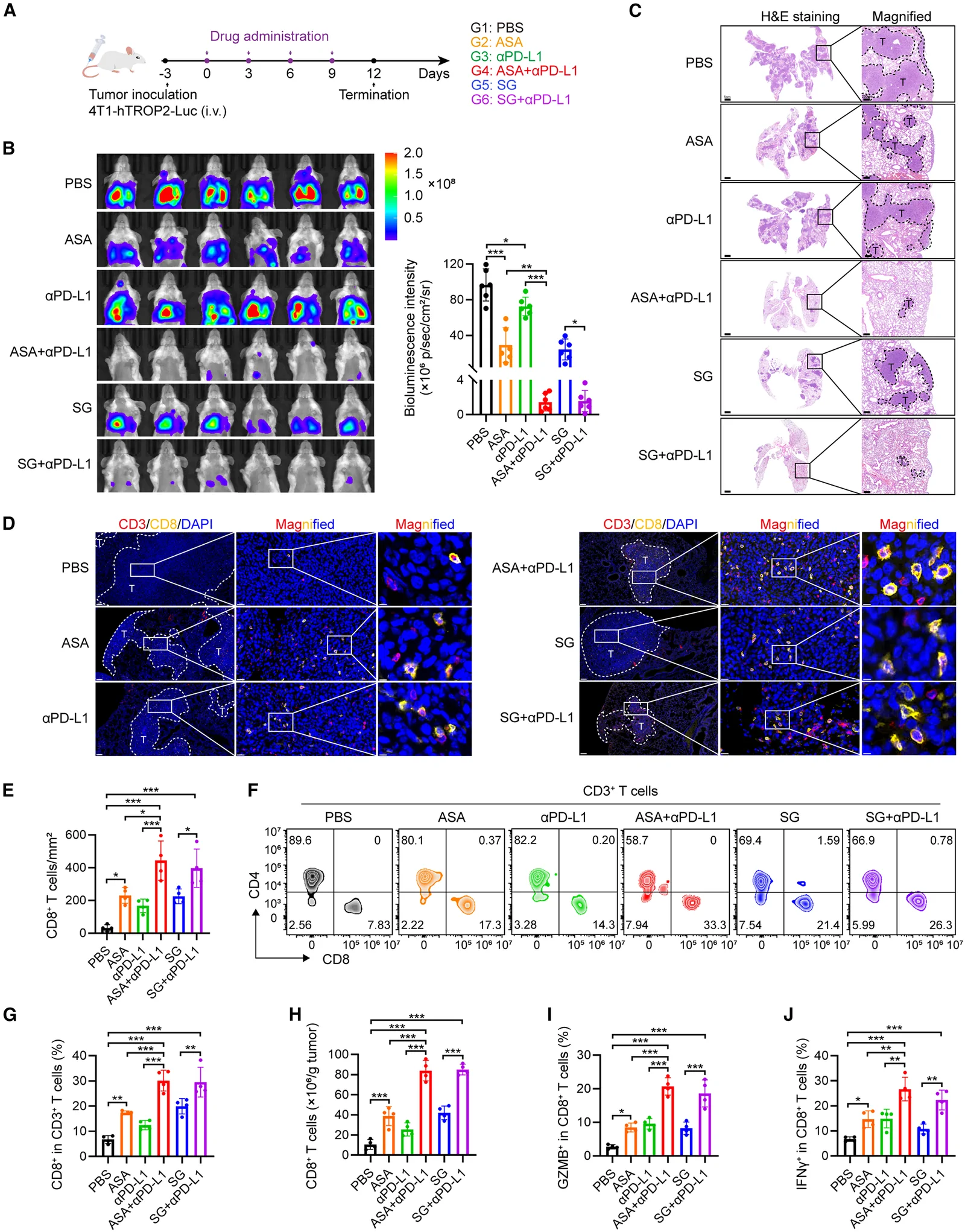
ASA and SG synergize with αPD-L1 to comparably suppress metastasis and potentiate CD8^+^ T cell infiltration and activation (A) Schematic illustrating the treatment regimens. Mice with lung metastasis were treated for 12 days with PBS (i.v., q3d), ASA (10 mg/kg, i.v., q3d), αPD-L1 (5 mg/kg, i.p., q3d), ASA (10 mg/kg, i.v., q3d) plus αPD-L1 (5 mg/kg, i.p., q3d), SG (10.27 mg/kg, i.v., q3d), or SG (10.27 mg/kg, i.v., q3d) plus αPD-L1 (5 mg/kg, i.p., q3d) (*n* = 6 mice per group). (B) Bioluminescent imaging (left) and quantification of bioluminescence intensity (right) of mice at the end of the study. (C) H&E staining of mouse lungs. Scale bars, 1 cm. (D and E) Representative immunofluorescence staining for CD3 and CD8 (D), and quantification of CD8^+^ T cells (E) in lung tissues from mice treated with different regimens. Scale bars: 100 μm and 20 and 5 μm (insets). (F and G) Flow cytometry quantification of CD8^+^ T cells among CD3^+^ T cells in lung tumor-infiltrating lymphocytes. (H) Quantification of the absolute number of CD8^+^ T cells in lung tumor-infiltrating lymphocytes. (I and J) Flow cytometry quantification of GZMB^+^ (I) and IFNγ^+^ (J) cells among CD8^+^ T cells in lung tumor-infiltrating lymphocytes. Data are presented as mean ± SD. ns, not significant; ∗*p* < 0.05, ∗∗*p* < 0.01, ∗∗∗*p* < 0.001 by one-way ANOVA. See also Figure S13.

Given the synergistic effect on tumor growth, ASA combined with αPD-L1 was further investigated to evaluate its efficacy in TNBC metastasis models compared with the SG and αPD-L1 cotreatment. SG, a potent ADC that shares the targeting moiety with ASA and utilizes the active metabolite of irinotecan (SN-38) as its cytotoxic payload, has been granted FDA approval for a specific-line treatment of TNBC.^40^ Its anti-tumor efficacy relies on the SAC-mediated TROP2 targeting, SN-38-induced cell apoptosis, and the subsequent induction of immunogenic cell death.^40^^,^^47^ In metastasis models, mice first received intravenous inoculation of 4T1-hTROP2-Luc cells, followed by administration of the indicated agents starting on day 3 post-inoculation and repeated every 3 days (Figure 6A). After 12 days of treatment, 10 mg/kg ASA and 10.27 mg/kg SG (with SAC equimolar to that in ASA) exerted comparable inhibitory effects on TNBC lung metastasis (69.7% and 74.9%), while 5 mg/kg αPD-L1 induced only moderate suppression (25.0%) (Figure 6B). Notably, concurrent treatments with αPD-L1 and either ASA or SG significantly enhanced anti-metastatic activity, increasing the inhibition rates by 28.8% and 23.4% compared with the corresponding monotherapies, revealing strong synergistic interactions, as further evidenced by H&E staining of lung lesions (Figures 6B and 6C). Thus, the robust anti-metastasis efficacy of these combinatorial regimens underscores the considerable translational potential of ASA for advanced TNBC treatment.

Based on these findings and ASA’s antitumor mechanisms, specifically targeting the BRD4/c-Myc/PD-L1 axis and inducing DNA repair deficiencies that theoretically contribute to reversing cytotoxic T cell exhaustion,^48^^,^^49^^,^^50^^,^^51^ we further assessed whether these regimens could enhance CD8^+^ T cell responses. Immunofluorescence of lung metastatic foci revealed increased CD8^+^ T cell infiltration in the ASA, αPD-L1, and SG monotherapy groups, with a more pronounced enhancement in combination groups (Figures 6D and 6E). This prominent increase in CD8^+^ T cells was validated by flow cytometry (Figures 6F–6H). We further examined granzyme B (GZMB) and interferon-γ (IFNγ) levels to evaluate whether the increased infiltration of CD8^+^ T cells is accompanied by their activation. Our results demonstrated that ASA, αPD-L1, or SG monotherapies upregulated GZMB and IFNγ levels in CD8^+^ T cells within metastatic lesions, with combination groups exerting a more pronounced effect (Figures 6I, 6J, S13A, and S13B). Overall, these findings illustrate that ASA combined with αPD-L1 mitigates TNBC metastasis and potently augments CD8^+^ T cell responses, an efficacy comparable to that of SG plus αPD-L1 cotreatment, thus establishing ASA as a promising agent for enhancing immunotherapeutic efficacy in TNBC.

### ASA promotes long-term anti-tumor immunological memory and synergizes with αPD-L1 for enhancement

**Figure 7:**
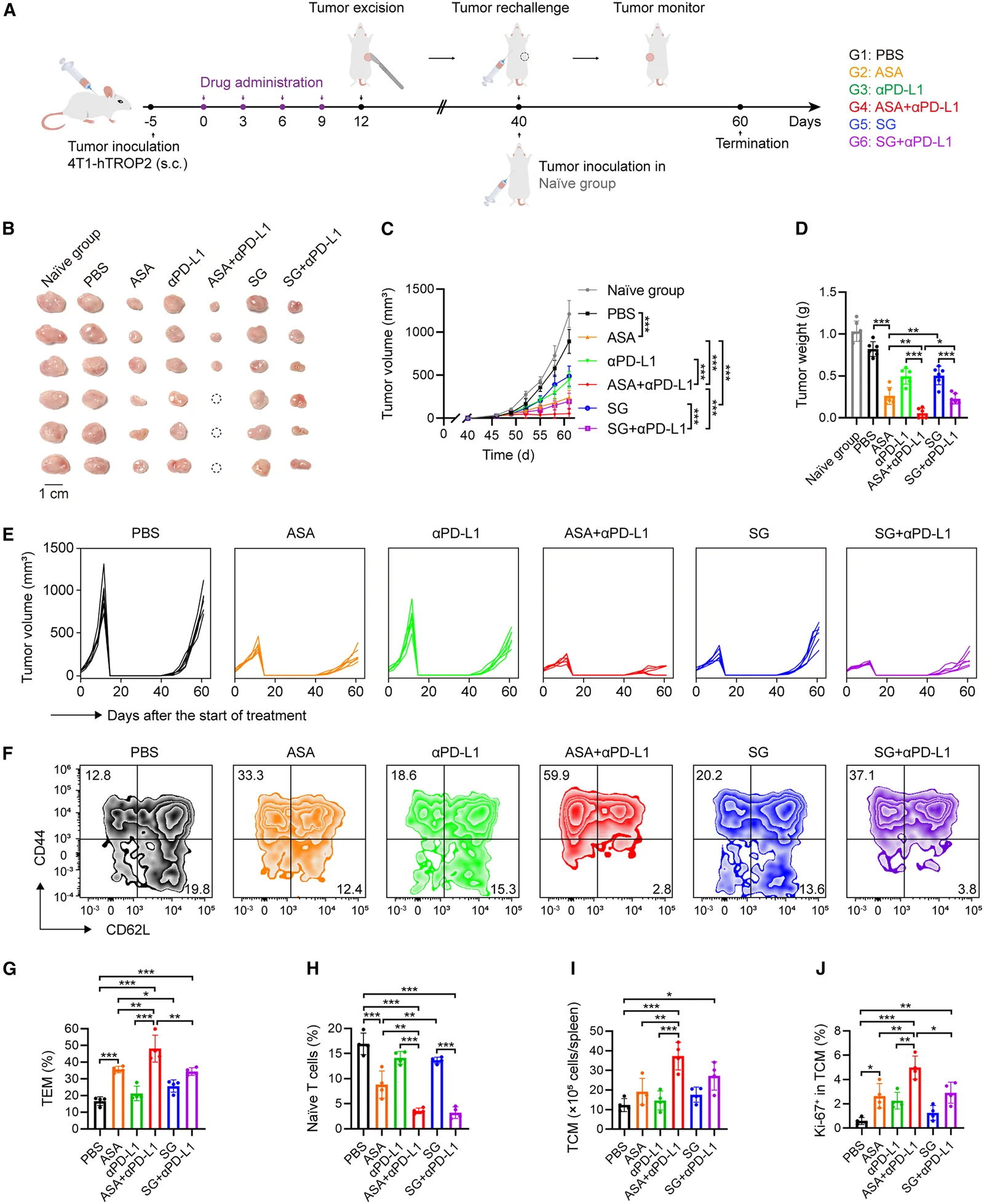
ASA promotes long-term anti-tumor immunological memory and synergizes with αPD-L1 for enhancement (A) Schematic illustrating long-term immunological memory evaluation. 4T1-hTROP2-bearing mice were treated with PBS (i.v., q3d), ASA (10 mg/kg, i.v., q3d), αPD-L1 (5 mg/kg, i.p., q3d), ASA (10 mg/kg, i.v., q3d) plus αPD-L1 (5 mg/kg, i.p., q3d), SG (10.27 mg/kg, i.v., q3d), or SG (10.27 mg/kg, i.v., q3d) plus αPD-L1 (5 mg/kg, i.p., q3d) (*n* = 6 mice per group). (B–D) Tumor image (scale bar, 1 cm) (B), tumor growth curves (C), and tumor weights (D). (E) Tumor growth curves for individual mice over 60 days. (F) Flow cytometry analysis of CD44 and CD62L expression in splenic CD8^+^ T cells. (G and H) Flow cytometry quantification of effector memory T cells (TEM) (G) and naive T cells (H) among splenic CD8^+^ T cells. (I) Quantification of the absolute number of central memory T cells (TCM) in splenic lymphocytes. (J) Flow cytometry quantification of Ki-67^+^ cell proportion among TCM in splenic lymphocytes. Data are presented as mean ± SD. ns, not significant; ∗*p* < 0.05, ∗∗*p* < 0.01, ∗∗∗*p* < 0.001 by two-way ANOVA (C) and one-way ANOVA (D and G–J). See also Figure S13.

To further evaluate the long-term protective effect of ASA against tumor rechallenge, mice were first implanted with 4T1-hTROP2 cells and then treated with the indicated agents for 12 days. Following treatment, the tumors were surgically resected, and tumor rechallenge was conducted 4 weeks post-surgery (Figure 7A). Analysis of tumor growth post-rechallenge revealed that mice treated with ASA displayed a robust immune memory effect, with a TGI rate of 72.9%, and this effect was even more pronounced in the group receiving the ASA plus αPD-L1 combination, achieving 93.9% TGI and a 50% tumor regression rate (Figures 7B–7E). In contrast, mice treated with SG and the SG plus αPD-L1 combination exhibited 45.2% and 77.8% TGI, respectively, upon rechallenge, reflecting a less-potent induction of immune memory. Persisting in the body for months, memory CD8^+^ T cells launch sustained tumor attacks post-surgery and foster enduring anti-tumor immunity to hinder tumor rechallenge.^52^ To clarify how ASA prevents tumor re-inoculation, we assessed memory T cell subsets in splenocytes, focusing on effector memory T cells (TEM) and central memory T cells (TCM). Flow cytometry analysis demonstrated a significant increase in the proportion of TEM (CD44^+^CD62L^−^) within the CD8^+^ T cell population of the ASA-treated group, indicating robust and effective activation of immunological memory (Figures 7F and 7G). Notably, a more pronounced effect was observed in mice receiving the combination regimen of ASA plus αPD-L1, whereas the SG plus αPD-L1 combination exhibited weaker efficacy in modulating TEM proportions (Figures 7F and 7G). Concomitantly, the proportion of naive T cells (CD44^−^CD62L^+^) was significantly reduced in the aforementioned treatment groups, consistent with the differentiation of naive T cells into memory T cell lineages during immune memory formation (Figure 7H). Moreover, the absolute count of TCM (CD44^+^CD62L^+^), together with the assessment of their proliferative potential, further corroborated that the ASA plus αPD-L1 combination markedly enhanced immunological memory activation by promoting memory T cell pool expansion (Figures 7I, 7J, and S13C). Collectively, these findings demonstrate that ASA induces superior long-term immunological memory compared with SG, while its synergy with αPD-L1 further potentiates this memory-driven antitumor response, thus affording robust protection against tumor rechallenge.

## Discussion

Breast cancer is the most prevalent malignancy and the leading cause of cancer-related deaths in women worldwide,^53^ with TNBC as the most aggressive subtype. Unfortunately, therapeutic options for TNBC remain scarce, and existing treatments often fail to meet the required efficacy and safety standards. Our team and other researchers have explored diverse therapeutic strategies for TNBC, including developing PROTACs to degrade c-Myc,^18^ screening high-efficiency aptamers for targeted delivery,^54^ and constructing nanoplatforms for synergistic therapy.^55^ Among these approaches, PROTACs, an emerging protein degradation technology, leverage the ubiquitin-proteasome system for sustained oncoprotein inhibition, providing promising avenues for treating cancers previously deemed “undruggable” or “incurable.” However, to date, no PROTAC-based agents have been developed that can selectively target TNBC cells to suppress disease progression. To address this critical gap in precision PROTAC-based therapies for TNBC, we designed and synthesized a TNBC-targeting antibody-PROTAC conjugate, termed ASA, in this work.

ASA utilizes SAC for the delivery of TROP2-positive TNBC cells and azobenzene linkers to enable hypoxia-sensitive release of the BRD4 PROTAC, ARV-771. Here, we demonstrated that ASA was selectively internalized by TNBC cells, where it was activated in response to hypoxia-triggered azoreductase to release the PROTAC payload. Dual dependence on TROP2 expression and hypoxic microenvironment confines cytotoxicity to tumor tissue and largely spares normal cells, in contrast to the unavoidable off-target toxicity of the ARV-771 monomer. *In vivo*, ASA rapidly accumulated in tumor sites, durably suppressed the BRD4/c-Myc/PD-L1 signaling axis, and elicited potent tumor inhibition while simultaneously awakening a therapeutically relevant antitumor immune response. In addition, ASA-induced DNA repair deficiencies suggest that TNBC may exhibit increased sensitivity to ICIs following ASA treatment. Combining ASA with αPD-L1 synergistically enhanced CD8^+^ T cell infiltration and activation, achieving rapid and robust tumor suppression, an effect comparable to the clinically investigated SG plus αPD-L1 cotreatment. Furthermore, this combination regimen elicited more robust long-term immunological memory, which effectively inhibited TNBC rechallenge and thereby established a therapeutic strategy with enduring clinical value for TNBC management.

Efforts to enhance PROTAC specificity are also being explored in other studies across various cancer types, with strategies including leveraging endogenous factors (e.g., pH-sensitive, cathepsin B, or GSH-cleavable linker),^56^^,^^57^^,^^58^ responding to exogenous stimuli (e.g., near-infrared light or ultrasound),^59^^,^^60^ or conjugating target-directed moieties (e.g., aptamers, peptides, or antibodies).^61^^,^^62^^,^^63^ Despite these advancements, TNBC-targeted PROTAC strategies remain limited due to their receptor-deficient profile, intricate TME, and distinctive signal transduction characteristics,^64^^,^^65^ necessitating multi-dimensional targeting strategies. Our ASA drug was designed by integrating the antibody-PROTAC model and TME-responsive features, enabling targeted modulation of the BRD4/c-Myc/PD-L1 signal and thus offering a strategy for antibody-PROTAC conjugates and PROTAC-based TNBC therapy. Specifically, compared with existing antibody-PROTAC conjugates that rely on conventional cleavable linkers and merely degrade the target protein,^23^^,^^24^^,^^25^^,^^63^ ASA features a hypoxia-responsive azobenzene linker that enables TME-specific payload release and achieves definitive suppression of the downstream axis, thereby exerting both direct antiproliferative effects and immunomodulatory activity. Relative to other PROTAC-based TNBC treatment strategies,^17^^,^^18^ ASA not only exhibited greater selectivity against tumor cells with less off-tumor proteolysis but also significantly enhanced CD8^+^ T cell responses and long-term immunological memory, whether in monotherapy or combined with αPD-L1, with the combinatorial regimen further achieving a 98.5% reduction in metastatic burden (Figure 6B) and a 93.9% inhibition of tumor rechallenge burden (Figure 7C).

This study introduces an alternative paradigm and paves the way for broader applications of PROTAC technology in TNBC treatment. The modular design of ASA could be adapted to target other “undruggable” oncoproteins in TNBC and other cancers.^17^^,^^18^^,^^66^^,^^67^ Future preclinical studies should explore more approaches in diverse TNBC models and TROP2-positive cancers. For example, incorporating dual-responsive linkers sensitive to multiple endogenous enzymes or exogenous stimuli could enhance payload release specificity, potentially in combination with photodynamic or sonodynamic therapies for precision in TNBC.^68^^,^^69^^,^^70^ Additionally, cell membrane-coated nanoparticles functionalized with TROP2-targeting ligands and encapsulating ARV-771 could further improve drug stability, targeting, and safety.^71^

Taken together, ASA combined a TROP2-targeted antibody with an azoreductase-cleavable linker to achieve precise intratumoral release of ARV-771, effectively suppressing BRD4/c-Myc/PD-L1 and DNA repair pathways to inhibit TNBC progression and enhance immunotherapeutic efficacy. This antibody-PROTAC conjugate is specifically designed for TNBC, extending the scope of current antibody-PROTAC approaches. By integrating antibody-guided tumor homing, hypoxia-responsive linker cleavage, and PROTAC-based precision, ASA enabled site-specific protein degradation within cancerous regions, which further synergized with αPD-L1 for enhanced antitumor immune response. This work thus opens a viable avenue for safe, effective, and durable TNBC management.

### Limitations of the study

A limitation of this study is the lack of global proteomic profiling comparing ASA- and ARV-771-treated cells under normoxic and hypoxic conditions. Such analysis would provide valuable insight into potential differences in off-target protein degradation and further clarify the hypoxia selectivity of ASA-induced BRD4 degradation, which remains an important direction for future investigation. Additionally, although preclinical models including PDX have demonstrated the antitumor efficacy and translational potential of ASA, they cannot fully recapitulate the complex tumor heterogeneity or long-term disease dynamics of TNBC in patients. Another potential limitation is that our study primarily focused on TROP2-high TNBC models, yet a small subset of TNBC patients exhibits low TROP2 expression or may develop acquired downregulation of TROP2 over the course of treatment. Therefore, well-designed clinical trials evaluating the safety, efficacy, and synergy of ASA with immunotherapies, along with biomarker-driven patient stratification, are essential for its clinical translation. Furthermore, given the specific overexpression of TROP2 in other malignancies (e.g., lung and cervical cancers), the application of ASA in these cancer types warrants further investigation.

## Resource availability

### Lead contact

Further information and requests for resources and reagents should be directed to and will be fulfilled by the lead contact, Xiaoxiang Guan (xguan@njmu.edu.cn).

### Materials availability

All materials generated in this study are available through the lead contact.

### Data and code availability

• The RNA-seq data generated for this study have been deposited at the Sequence Read Archive database of NCBI. Accession numbers are listed in the key resources table.
• This paper does not report original code.
• Any additional information required to reanalyze the data reported in this paper is available from the lead contact upon request.

## Acknowledgments

This work was supported by the State Key Program of the National Natural Science Foundation of China (82430093) and the 10.13039/501100001809National Natural Science Foundation of China (22322703 and 22577050).

## Author contributions

X.G. and J.L. conceived the study, secured funding, and supervised the research implementation; Y.G. and C.L. designed the methodology, organized the data, and contributed to manuscript writing and refinement; Y.G., C.L., T.X., and Y. Zhao performed *in vivo* and *in vitro* experiments and collected data; D.W. and Y. Zhu collected patient tumor tissues and clinical data; Y.G., C.L., X.W., J.C., J.L., and X.G. reviewed and revised the manuscript.

## Declaration of interests

The authors declare no competing interests.

## STAR★Methods

### Key resources table

**Table:** 

REAGENT or RESOURCE	SOURCE	IDENTIFIER
**Antibodies**		

Rabbit monoclonal anti-TROP2	Cell Signaling Technology	Cat#47866; RRID: AB_2938529
Mouse monoclonal anti-β-Actin	Cell Signaling Technology	Cat#58169; RRID: AB_2750839
HRP-linked anti-rabbit IgG	Cell Signaling Technology	Cat#7074; RRID: AB_2099233
HRP-linked anti-mouse IgG	Cell Signaling Technology	Cat#7076; RRID: AB_330924
Mouse monoclonal anti- Phospho-Histone H2A.X (Ser139)	Cell Signaling Technology	Cat#80312; RRID: AB_2799949
Rabbit polyclonal anti-NQO1	Proteintech	Cat#11451-1-AP; RRID: AB_2298729
Rabbit polyclonal anti-BRD4	Proteintech	Cat#28486-1-AP; RRID: AB_2918170
Rabbit monoclonal anti-ATP1A1	Proteintech	Cat#14418-1-AP; RRID: AB_2227873
Mouse monoclonal anti-*c*-Myc	Proteintech	Cat# 67447-1-Ig; RRID: AB_2882681
Mouse monoclonal anti-PD-L1	Proteintech	Cat#66248-1-Ig; RRID: AB_2756526
Sacituzumab	MedChemExpress	Cat#HY-P99045; RRID: AB_3694490
Human IgG1 kappa	MedChemExpress	Cat#HY-P99001; RRID: AB_3739867
InVivoMAb anti-mouse PD-L1	BioXcell	Cat#BE0101; RRID: AB_10949073
Purified anti-mouse CD16/32 Ab	Biolegend	Cat#101302; RRID: AB_312801
CD45 Monoclonal Antibody (30-F11), Alexa Fluor™ 700	Thermo Fisher Scientific	Cat#56-0451-82; RRID: AB_891454
CD3 Monoclonal Antibody (17A2), eFluor™ 506	Thermo Fisher Scientific	Cat#69-0032-82; RRID: AB_2637122
CD4 Monoclonal Antibody (GK1.5), FITC	Thermo Fisher Scientific	Cat#11-0041-81; RRID: AB_464891
CD8a Monoclonal Antibody (53–6.7), PE-Cyanine7	Thermo Fisher Scientific	Cat#25-0081-82; RRID: AB_469584
IFN gamma Monoclonal Antibody (XMG1.2), eFluor™ 450	Thermo Fisher Scientific	Cat#48-7311-82; RRID: AB_1834366
CD44 Monoclonal Antibody (IM7), PE	Thermo Fisher Scientific	Cat#12-0441-81; RRID: AB_465663
CD62L (L-Selectin) Monoclonal Antibody (MEL-14), APC	Thermo Fisher Scientific	Cat#17-0621-81; RRID: AB_469409
Ki-67 Monoclonal Antibody (SolA15), eFluor™ 450	Thermo Fisher Scientific	Cat#48-5698-82; RRID: AB_11149124
Anti-Ki67 Rabbit pAb	Servicebio	Cat#GB111141; RRID: AB_3096315
Recombinant Anti-CD3 antibody (Mouse mAb)	Servicebio	Cat#GB15014; RRID: AB_3741677
Recombinant Anti-CD8 alpha antibody (Rabbit mAb)	Servicebio	Cat#GB15068; RRID: AB_3246431
Cy3-conjugated goat anti-mouse IgG	Servicebio	Cat#GB21301; RRID: AB_2923552
Cy5-conjugated goat anti-rabbit IgG	Servicebio	Cat#GB27303; RRID: AB_2905513
CoraLite® Plus 594-Goat Anti-Mouse Recombinant Secondary Antibody (H + L)	Proteintech	Cat#RGAM004; RRID: AB_3073502
CoraLite Plus 488-Goat Anti-Rabbit Recombinant Secondary Antibody (H + L)	Proteintech	Cat#RGAR002; RRID: AB_3073506
Rabbit polyclonal anti-TROP2	Proteintech	Cat#27360-1-AP; RRID: AB_2918122
Rabbit polyclonal anti-ER	Proteintech	Cat#21244-1-AP; RRID: AB_11042600
Rabbit polyclonal anti-PR	Proteintech	Cat#25871-1-AP; RRID: AB_2880277
Rabbit polyclonal anti-HER2/ErbB2	Proteintech	Cat#18299-1-AP; RRID: AB_2099264

**Biological samples**		

Human TNBC biopsy tissue	Jiangsu Provincial People’s Hospital	N/A

**Chemicals, peptides, and recombinant proteins**		

TRIzol	Invitrogen	Cat#15596018
RIPA buffer	Beyotime	Cat#P0013C
PMSF and phosphatase inhibitor	Beyotime	Cat#P1045
Bovine Serum Albumin	Biosharp	Cat#BS114
Hoechst 33342	Beyotime	Cat#C1028
Antifade mounting medium (with DAPI)	Beyotime	Cat#P0131
DMSO	Beyotime	Cat#ST038
Lipofectamine 3000 transfection reagent	Thermo Fisher Scientific	Cat#L3000015
Fixable Viability Dye eFluor™ 780	Thermo Fisher Scientific	Cat#65-0865-14
Puromycin	Beyotime	Cat#ST551
DNase I	Beyotime	Cat#D7076
Collagenase Type IV	Sigma	Cat#C4-28
Percoll	Sigma	Cat#P4937
Cell Stimulation Cocktail (plus protein transport inhibitors) (500×)	Thermo Fisher Scientific	Cat#00-4975-93
Foxp3/Transcription Factor Staining Fixation/Permeabilization Buffer	Thermo Fisher Scientific	Cat#00-5523-00
Camptothecin	MedChemExpress	Cat#HY-16560
Dicoumarol	MedChemExpress	Cat#HY-N0645
MG-132	MedChemExpress	Cat#HY-13259
D-Luciferin, Potassium Salt	GoldBio	Cat#LUCK
Opti-MEM™	Gibco	Cat#31985070
Fetal Bovine Serum	Gibco	Cat#A5256701
DMEM	Gibco	Cat#11995073
RPMI 1640 Medium	Gibco	Cat#11875093

**Critical commercial assays**		

BCA Protein Assay Kit	Beyotime	Cat#P0010
ECL Kit	Biosharp	BL520A
Annexin V-FITC/PI Apoptosis Detection Kit	Vazyme	A211
HiScript III All-in-one RT SuperMix	Vazyme	Cat#R433-01
ChamQ Universal SYBR qPCR Master Mix	Vazyme	Cat#Q312-02
Cell Counting Kit-8	MedChemExpress	Cat#HY-K0301

**Deposited data**		

RNA sequence	This paper	SRA: PRJNA1357832

**Experimental models: Cell lines**		

Human: HCC1806	ATCC	RRID: CVCL_1258
Human: MDA-MB-468	ATCC	RRID: CVCL_0419
Human: T-47D	ATCC	RRID: CVCL_0553
Human: MCF7	ATCC	RRID: CVCL_0031
Human: MCF10A	ATCC	RRID: CVCL_0598
Mouse: 4T1	ATCC	RRID: CVCL_0125
Mouse: 4T1-hTROP2	This paper	N/A

**Experimental models: Organisms/strains**		

Mouse: BALB/cJGpt	GemPharmatech	Cat#N000020; RRID: IMSR_GPT:N000020
Mouse: NOD/ShiLtJGpt-Prkdc^em26Cd52^/Gpt	GemPharmatech	Cat#T001492; RRID: IMSR_GPT: T001492

**Oligonucleotides**		

siRNA sequences	This paper	See Table S3
qRT-PCR Primer sequence	This paper	See Table S4

**Recombinant DNA**		

pLV-CMV-hTACSTD2-EF1A-Puro	GeneChem	N/A
pcDNA3.1-*c*-Myc	GeneChem	N/A

**Software and algorithms**		

GraphPad Prism 9.0	GraphPad Software, Inc.	https://www.graphpad.com/
R 4.2	N/A	https://www.r-project.org/
FlowJo 10.10.0	BD Biosciences	https://www.flowjo.com/
ImageJ 1.53	NIH	https://imagej.nih.gov/ij/

### Experimental model and study participant details

#### Cell lines

The human breast cancer cell lines HCC1806, MDA-MB-468, T-47D, MCF7, normal human mammary epithelial cell line MCF10A, and murine mammary carcinoma cell line 4T1 were obtained from ATCC. All cell lines were used within 25 passages, authenticated by the short tandem repeat method, and confirmed mycoplasma-free. HCC1806, T-47D, and 4T1 were cultured in RPMI-1640 (Gibco), while MDA-MB-468 and MCF7 were maintained in DMEM (Gibco); both media were supplemented with 10% FBS (Gibco). MCF10A was cultured in DMEM/F12 supplemented with 5% horse serum (HS), 20 ng/mL epidermal growth factor (EGF), 0.5 μg/mL hydrocortisone, 10 μg/mL insulin, 1% non-essential amino acids (NEAA), and 1% penicillin/streptomycin. Cells under normoxic conditions were incubated at 37°C in a humidified atmosphere of 20% O_2_, 5% CO_2_, and balance nitrogen. For the hypoxic group, cells were cultured in a specialized low-oxygen incubator at 37°C, with a humidified atmosphere maintained at 1% O_2_, 5% CO_2_, and balance nitrogen.

#### Patient-derived materials

All human sample procedures were approved by the Ethics Committee of The First Affiliated Hospital of Nanjing Medical University (Approval No. 2025-SR-887) and performed in accordance with relevant ethical guidelines. Written informed consent was obtained from the donor patient. Fresh tumor tissue was obtained from a needle biopsy of a primary TNBC tumor in a 41-year-old treatment-naïve female patient and immediately placed in DMEM medium. The tissue was then rinsed with antibiotic-supplemented DMEM, and necrotic areas, blood clots, and adipose tissue were carefully removed. The remaining tumor tissue was then sectioned into small fragments of approximately 2 mm in diameter, and subcutaneously implanted into 6-week-old NCG mice under anesthesia following standard procedures. When tumors reached approximately 1,000 mm^3^, they were excised and re-implanted into new recipients. Histopathologic analysis confirmed that the patient-derived tumor xenograft (PDX) model recapitulated the TROP2-high TNBC phenotype. PDX tumors at passage 2 (P2) were used for the subsequent animal experiments.

#### Animals

All *in vivo* experiments adhered to the Animal Research: Reporting of *In Vivo* Experiments (ARRIVE) guidelines and were approved by the Institutional Animal Care and Use Committee of Nanjing Medical University (IACUC-2507008). Six-week-old female BALB/c mice (immunocompetent) and NCG mice (immunodeficient) purchased from GemPharmatech Co., Ltd. were healthy and maintained under Specific Pathogen-Free conditions with a 12 h light/12 h dark cycle, with free access to standard chow and sterile water. For subcutaneous tumor models, 2 × 10^6^ cancer cells were subcutaneously injected into the left flank of each mouse. Mice were randomized into the respective treatment groups 5 days post-inoculation, and tumor volumes (calculated as length × width^2^/2) as well as body weights were recorded every 3 days. At study termination, tumors and major organs were excised for histopathological and molecular analyses, and peripheral blood was collected to measure serum biochemical indicators. For lung metastasis models, 5 × 10^5^ cancer cells were injected intravenously via the tail vein of each mouse. Mice were randomized into the respective treatment groups 3 days post-inoculation, and at study termination, bioluminescence intensity (average radiant efficiency, photons/second/cm^2^/steradian) of lung metastasis was quantified via an IVIS Lumina III Imaging System, followed by lung dissection for further analysis. For long-term immunological memory models, left-side tumor-bearing mice received the indicated treatments for 12 days, followed by tumor excision. After 4 weeks, tumor rechallenge was performed on the right side, and mice were monitored for 20 days. Naive group mice were inoculated with tumors on the right side on day 40 and monitored for 20 days. At study termination, tumors and spleens were harvested for further analysis. For PDX model, fresh tumor tissue obtained from a needle biopsy of a primary TNBC was implanted into 6-week-old NCG mice under anesthesia. Once the tumors reached approximately 1,000 mm^3^, they were excised and re-implanted into new recipients. Second-passage PDX tumors (P2) were randomized into treatment groups when they reached approximately 1,000 mm^3^. At study termination, tumors were excised for histopathological analyses.

### Method details

#### Stability and ARV-771 release assays

To evaluate the serum stability of ASA conjugates, the conjugates were dissolved in DMEM supplemented with 20% FBS to a final concentration of 0.2 mg/mL and incubated at 37°C for 0–96 h. At the indicated time points, aliquots were collected, centrifuged to precipitate proteins, and analyzed by high-performance liquid chromatography (HPLC). For evaluating ASA stability in the presence of various biological analytes, ASA was incubated with CaCl_2_, Cys, Glu, H_2_O_2_, KCl, MgCl_2_, NaCl, NaClO, NADPH, or Ser under 20% O_2_ for 24 h, while a control group containing azoreductase was incubated under 1% O_2_ for 24 h. All samples were quenched with ice-cold methanol, centrifuged, and analyzed by HPLC. To investigate *in vitro* ARV-771 release from ASA under hypoxic or normoxic conditions, NADPH (100 mM) and azoreductase at different concentrations were added to a PBS solution of ASA (1 mM) containing 1% DMSO (v/v) maintained under 1% O_2_ or 20% O_2_. The mixture was incubated at 37°C, with aliquots collected at indicated time points, quenched with acetonitrile, centrifuged, and analyzed by HPLC. To assess *in vivo* ARV-771 release, tumor tissues, liver, and plasma were collected at 24 and 72 h post-administration. Tissues were homogenized, and all samples were precipitated with acetonitrile, centrifuged to remove proteins, and analyzed by HPLC.

#### Lentiviral transduction and cell transfection

Lentiviral vectors encoding human *TACSTD2 (hTACSTD2)* and a negative control (empty vector) were obtained from GeneChem. Stable 4T1 cell line overexpressing hTROP2 (4T1-hTROP2) and control cell line (4T1-NC) were generated via lentiviral transduction followed by antibiotic selection, according to the manufacturer’s instructions. Stable hTROP2 expression was verified by western blot analysis. For c-Myc overexpression, TNBC cells were transfected with a c-Myc overexpression plasmid (GeneChem) or empty vector control using Lipofectamine 3000 (Thermo Fisher Scientific), and overexpression was confirmed by western blot analysis 48 h post-transfection. For BRD4 knockdown, cells were transfected with BRD4-targeting siRNA or control siRNA using Lipofectamine 3000, and knockdown efficiency was verified. Sequences of siRNA used were detailed in Table S3.

#### Western blot

Cellular and tissue proteins were extracted by lysing or homogenizing in RIPA buffer (with 1% PMSF and protease inhibitor cocktail) on ice, followed by centrifugation to collect supernatant. Proteins were separated by 6%–10% SDS-PAGE and transferred onto PVDF membranes (Millipore). Membranes were blocked with 5% non-fat dry milk in TBST for 2 h at room temperature, then incubated with primary antibodies overnight at 4°C, followed by HRP-conjugated secondary antibodies for 1 h at room temperature. Bands were visualized using an ECL kit (Biosharp) and quantified via ImageJ. Antibodies used in this study are detailed in the key resources table.

#### Quantitative real-time PCR (qRT-PCR)

Total RNA was extracted using TRIzol reagent (Invitrogen). Reverse transcription and quantitative PCR were performed using HiScript III All-in-one RT SuperMix (Vazyme) and ChamQ Universal SYBR qPCR Master Mix (Vazyme), respectively, on a QuantStudio 7 Pro Real-Time PCR System (Applied Biosystems). Relative gene expression was calculated using the comparative Ct (ΔΔCt) method, with primer sequences listed in Table S4.

#### Binding affinity assessment

Single-cell suspensions were pretreated with Fc receptor-blocking solution (BioLegend) in PBS for 10 min at room temperature, and then incubated with a fluorescently labeled drug at increasing concentrations in FACS buffer for 20 min at 4°C. After two washes with FACS buffer to remove unbound drug, samples were analyzed using CytoFlex (Beckman Coulter). Data were processed with FlowJo (version 10.10.0), and mean fluorescence intensity (MFI) was quantified to assess binding affinity.

#### Confocal fluorescence imaging

To assess cellular uptake, HCC1806, MDA-MB-468, 4T1-hTROP2, and 4T1-NC cells were seeded in 35-mm glass-bottom dishes and cultured overnight at 37°C in a humidified 5% CO_2_ atmosphere. Cells were pre-incubated with 20 μM Cy5-labeled ASA for 20 min at 4°C to allow initial binding, then incubated at 37°C for 0, 0.5, or 2 h to assess uptake. After incubation, cells were gently washed three times with cold PBS to remove unbound dye. Nuclei were stained with Hoechst 33342 for 5 min at room temperature, followed by three additional PBS washes to remove excess stain. Fluorescence images were acquired using a Leica STELLARIS 5 laser-scanning confocal microscope.

#### Cell viability and apoptosis assay

HCC1806, MDA-MB-468, T-47D, MCF7, 4T1-NC, 4T1-hTROP2, and MCF10A cells were seeded in 96-well plates (2,000 cells/well: HCC1806, 4T1-NC, 4T1-hTROP2; 4,000 cells/well: MDA-MB-468, T-47D, MCF7; 6,000 cells/well: MCF10A) and pre-incubated under 1% O_2_ or 20% O_2_ for 24 h. Once the 48-h treatments under corresponding oxygen conditions were complete, cell viability was assessed using a CCK-8 kit (MedChemExpress). To evaluate cell apoptosis, Annexin V-FITC/PI flow cytometry was performed. HCC1806, MDA-MB-468, and 4T1-hTROP2 cells were seeded in 12-well plates and pre-incubated under 1% O_2_ or 20% O_2_ for 24 h. After 24-h treatments under corresponding oxygen conditions, cells were harvested by trypsinization, washed twice with PBS, and stained using an Annexin V-FITC/PI kit (Vazyme) according to manufacturer’s instructions. Apoptotic cells were quantified by flow cytometry and analyzed via FlowJo.

#### Wound healing and transwell assay

Wound healing assays were performed by seeding HCC1806, MDA-MB-468, and 4T1-hTROP2 cells in 12-well plates, pre-incubating for 24 h and treating them with indicated drugs for another 24 h under 20% O_2_ or 1% O_2_, then creating a linear scratch using a 10 μL pipette tip, washing with PBS to remove debris, and incubating in fresh 1% FBS-containing medium for 36 h (HCC1806 and MDA-MB-468) or 18 h (4T1-hTROP2). Wound closure was imaged at 0 h and endpoint using an inverted microscope. Wound area was measured via ImageJ, and healing rate was calculated as [(Initial wound area − Endpoint wound area)/Initial wound area] × 100 (%). For transwell assays, cells were seeded in 12-well plates, pre-incubated for 24 h and treated with indicated drugs for another 24 h under 20% O_2_ or 1% O_2_, then trypsinized and seeded into the upper chambers of Matrigel-precoated transwell inserts (8 μm pore size) at proper densities (4 × 10^4^ cells/insert: HCC1806, MDA-MB-468; 2 × 10^4^ cells/insert: 4T1-hTROP2) in serum-free medium (lower chambers with 10% FBS medium), followed by incubation for 24–48 h. Non-invading cells on the upper insert surface were removed with a cotton swab. Invading cells on the lower surface were fixed with 4% paraformaldehyde, stained with 0.1% crystal violet, imaged via an inverted microscope, and quantified (number or covered area) using ImageJ.

#### Fluorescence imaging and immunofluorescence staining

Whole-body fluorescence imaging of 4T1-hTROP2-bearing mice treated with Cy5-labeled drugs was performed using an IVIS Lumina III Imaging System (Revvity) at excitation 640 nm and emission 670 nm at the at the indicated time points following administration. *Ex vivo* fluorescence imaging of resected tissues was also conducted 48 h post-treatment. Fluorescence intensity (FI) was quantified as total radiant efficiency (photons/second/cm^2^/steradian). Tumors from mice treated with Cy5-labeled drugs were snap-frozen, sectioned, and stained with DAPI for fluorescence imaging. For immunofluorescence analysis of lung metastases, tissues were fixed, paraffin-embedded, sectioned, and subjected to antigen retrieval after deparaffinization. Sections were blocked, incubated with anti-CD3 and anti-CD8 primary antibodies, followed by Cy3-and Cy5-conjugated secondary antibodies, and counterstained with DAPI prior to imaging using a Nikon Eclipse C1 fluorescence microscope.

#### RNA sequencing analysis

Total RNA was extracted using TRIzol reagent (Invitrogen), with concentration and quality assessed by NanoDrop (Thermo Fisher) and integrity confirmed by denaturing agarose gel electrophoresis. Poly(A) mRNA was purified, fragmented, and reverse-transcribed to cDNA, followed by second-strand DNA synthesis, end repair, A-tailing, adapter ligation, and size selection. PCR amplification was performed to generate the final cDNA library (average insert size: 300 ± 50 bp), which was sequenced using the Illumina NovaSeq 6000 (Illumina). Raw sequencing data were quality-controlled, and clean reads were mapped to the reference genome. The identification of differentially expressed genes (DEGs) was performed using the edgeR package in R software, and gene set enrichment analysis (GSEA) was conducted with the clusterProfiler package based on MSigDB Collections, with relevant visualizations generated using ggplot2.

#### Hematoxylin and eosin (H&E) and immunohistochemical (IHC) staining

Experimental mouse tissues were fixed in 4% paraformaldehyde, embedded in paraffin, and sectioned. For H&E staining, sections were deparaffinized, rehydrated, stained with hematoxylin-eosin, dehydrated, and mounted. For IHC staining, sections were deparaffinized, rehydrated, and subjected to antigen retrieval. Endogenous peroxidase was quenched with 3% hydrogen peroxide for 20 min, followed by PBS washes. Sections were blocked with 3% BSA, then incubated overnight at 4°C with primary antibodies. Afterward, sections were incubated with secondary antibodies for 1 h at room temperature, visualized with DAB, counterstained with hematoxylin, dehydrated, and mounted for microscopy. IHC staining was quantified by the histochemistry score‌ (H-score) using ImageJ. Antibodies used in this study are detailed in the key resources table.

#### Flow cytometric analysis of immune cells

Tumor tissues were mechanically dissociated into small fragments and incubated with enzyme digestion solution at 37°C for 30-60 min to obtain single-cell suspensions. Spleens were ground to prepare single-cell suspensions. Lymphocytes were isolated via Percoll density gradient centrifugation (Sigma) and treated with a stimulation/blocking agent (eBioscience, Thermo Fisher Scientific). Single-cell suspensions were pre-treated with Fc receptor-blocking solution, then stained with Fixable Viability Dye (eBioscience) on ice for 30 min, followed by staining with diluted fluorochrome-conjugated antibodies on ice for another 30 min. For intracellular staining, cells were fixed and permeabilized using the Intracellular Fixation/Permeabilization Kit (eBioscience), followed by intracellular dye staining on ice for 30 min. Flow cytometry was performed on CytoFlex, and data were analyzed with FlowJo, with gating strategies detailed in Figure S14. Fluorescent dye-conjugated antibodies used in this study are detailed in the key resources table.

#### Antibody modification and conjugation

Humanized anti-TROP-2 antibody Sacituzumab (SAC) in PBS was treated with varying equivalents of DBCO-NHS (5 mM stock) for 12 h at room temperature to afford DBCO-SAC conjugates with different DAR. Specifically, treatment with approximately 1, 2, or 5 equivalents of DBCO-NHS per antibody yielded DAR values of approximately 2, 5, and 8, respectively. Humanized IgG1 kappa was treated under the same conditions with approximately 50 equivalents of DBCO-NHS to yield DBCO-IgG with a DAR of approximately 5. In all cases, excess small molecules were removed by ultrafiltration (MWCO 10 kDa) against PBS for five cycles. The resulting DBCO-modified antibodies were then incubated with an excess of *n*-7 (approximately 5 equivalents) in PBS at 37°C for 4 h. After centrifugation, the supernatants were collected and stored at −80°C until further use. The product derived from DBCO-SAC (DAR ≈5) is designated as ASA, while the product from DBCO-IgG (DAR ≈5) is designated as IgG-ARV-771. In addition, conjugation of DBCO-SAC (DAR ≈5) with c-2 under identical conditions afforded NSA. The synthesis of *n*-7 and c-2 is shown in Figure S15. For *in vivo* imaging studies, both ASA and IgG-ARV-771 were labeled with Cy5-NHS in PBS at room temperature for 2 h, and excess dye was removed by ultrafiltration (MWCO 10 kDa) (Figure S16).

#### SDS-PAGE analysis

ASA samples (10 μg total protein per lane) were mixed with reducing or non-reducing loading buffer and heated at 95°C for 5 min. Proteins were separated on 4–12% Bis-Tris gradient polyacrylamide gels at a constant voltage of 100 V using 1× running buffer, alongside pre-stained protein standards (10–250 kDa). Following electrophoresis, the gel was stained with Coomassie Blue and imaged using the ChemiDoc MP system (Bio-Rad) (Figure S17).

#### Chemical analysis and instrumentation

All chemicals and solvents were purchased from MCE or Sigma-Aldrich. All aqueous solutions were treated with diethyl pyrocarbonate (DEPC) prior to use. ESI-HRMS and ESI-LCMS were conducted on a Q Exactive (Thermo Fisher) and an LCMS-2020 (SHIMADZU), respectively. LC-MS was carried out on a Xevo G2-XS QTof mass spectrometer coupled to an Acquity UPLC system equipped with an Acquity UPLC Protein BEH C4 column (1.7 μm, 2.1 × 50 mm) (Figure S18). ^1^H NMR and ^13^C NMR spectra were recorded on Bruker Avance III 400 or 600 MHz spectrometers, with chemical shifts (δ) reported in ppm relative to residual solvent peaks (Figures S19 and S20). HPLC analysis was performed on a Thermo Scientific Dionex Ultimate 3000 system using CH_3_CN/H_2_O (containing 0.1% CF_3_COOH) as eluents.

### Quantification and statistical analysis

Two-tailed unpaired Student’s *t* test and ANOVA were employed to determine statistical differences between two groups and among multiple groups, respectively. A *p*-value less than 0.05 was considered statistically significant (ns, not significant, ∗*p* < 0.05, ∗∗*p* < 0.01, ∗∗∗*p* < 0.001). All experiments were independently repeated at least three times. Statistical analyses were performed using GraphPad Prism 9.0 (GraphPad Software, San Diego, CA, USA) and R version 4.2 (R Foundation for Statistical Computing, Vienna, Austria).

## Supplemental information

Document S1. Figures S1–S20 and Tables S1–S4

Document S2. Article plus supplemental information
