# Supplementary material for: An antibody-PROTAC conjugate targets BRD4/c-Myc/PD-L1 to enhance immunotherapy efficacy in triple-negative breast cancer
Source: Cell Rep Med. 2026 Jul 29;7(8):102948. doi: 10.1016/j.xcrm.2026.102948 (PMC13522800; doi:10.1016/j.xcrm.2026.102948)
Supplement: Supplementary file 1 [file mmc1.pdf]

Cell Reports Medicine, Volume *7*

Supplemental information

An antibody-PROTAC conjugate targets BRD4/c-Myc/PD-L1 to enhance immunotherapy efficacy in triple-negative breast cancer

Yunru Gu, Chen Li, Yuhan Zhao, Tianrui Xu, Ye Zhu, Dandan Wang, Xiaoning Wang, Jiaxuan Chen, Jinbo Li, and Xiaoxiang Guan

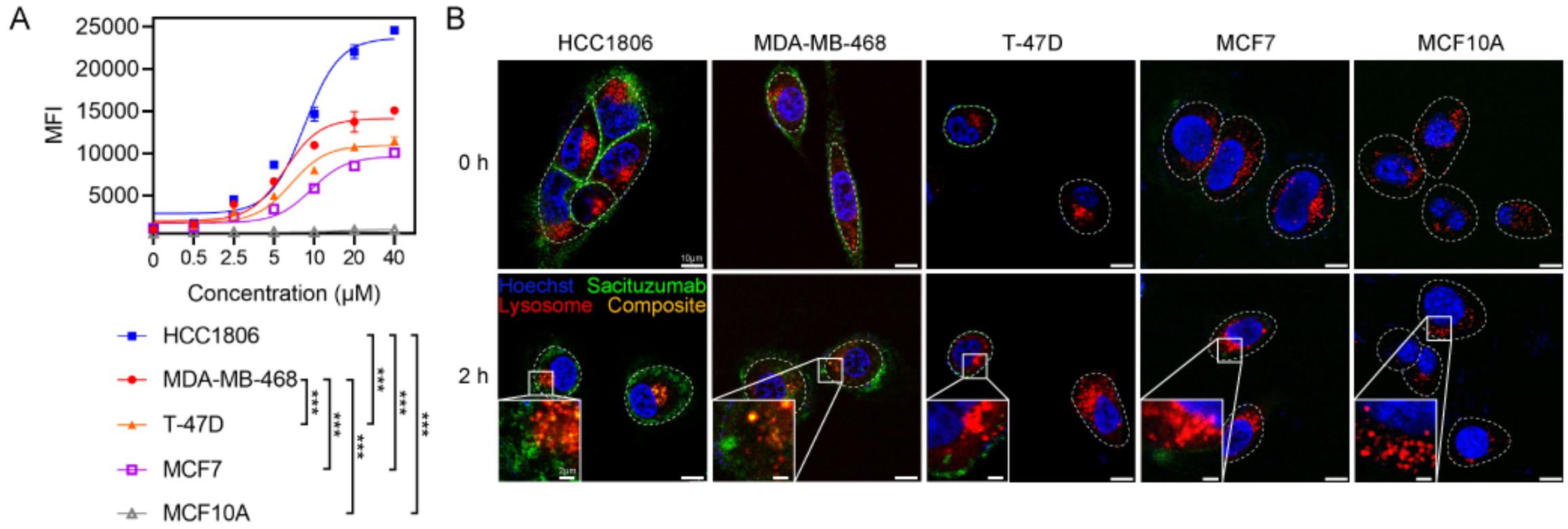

Figure S1 Binding affinity and internalization efficiency of SAC (Sacituzumab) in different cell types. Related to Figure 1.

(A) Quantification of MFI assessing SAC binding affinity to TROP2 in TNBC cells (HCC1806, MDA-MB-468), ER- and PR-positive breast cancer cells (T-47D, MCF7), and normal human mammary epithelial cells (MCF10A).

(B) Cellular internalization and lysosome colocalization of SAC in different cell lines following 20-min pre-incubation at 4℃ and subsequent 37℃ incubation for 0 h or 2 h. SAC was labelled with FITC, lysosome was labelled with LysoTracker Red, and nucleus was labeled with Hoechst 33342. Scale bars, 10 μm (insets, 2 μm).

Data are presented as mean ± SD. ns, not significant; **p* < 0.05, ***p* < 0.01, ****p* < 0.001 by two-way ANOVA.

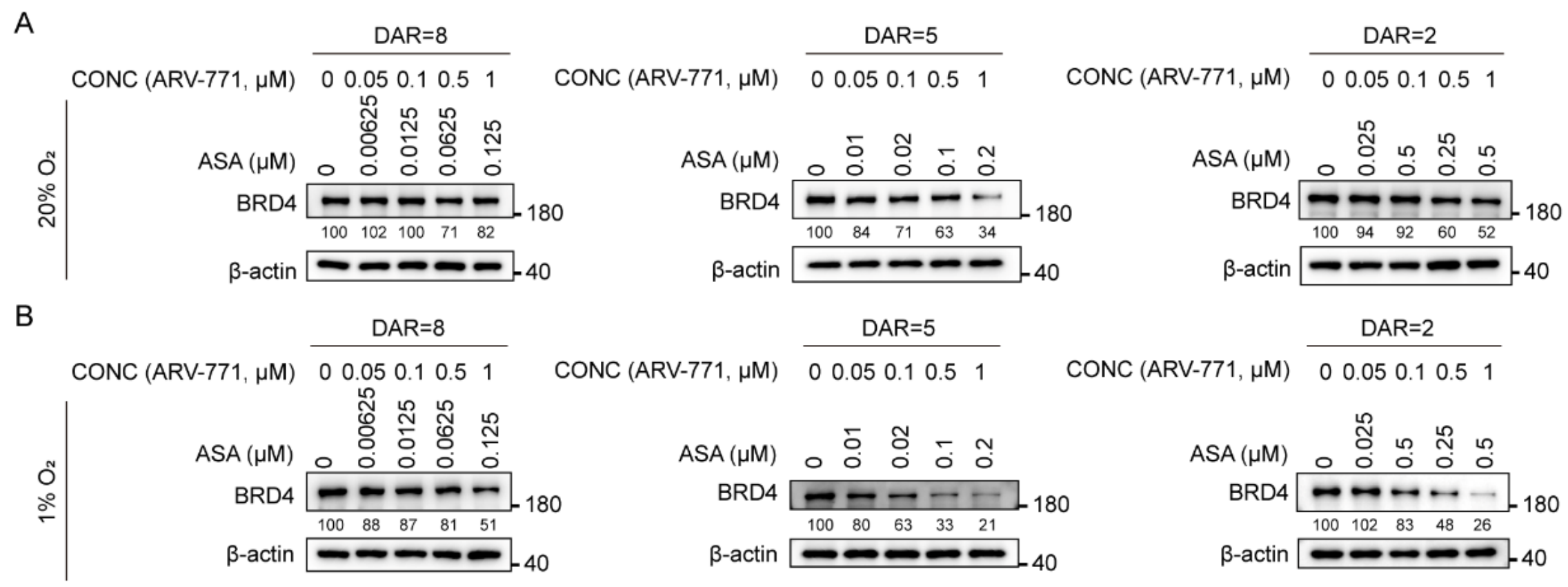

Figure S2 Western blot analysis of BRD4 in HCC1806 cells treated with a concentration gradient of ASA with different drug-to-antibody ratios (DARs) at the same PROTAC dose under 20% O_2 (A) and 1% O_2 (B). Related to Figure 1. Concentration (CONC) of ARV-771 was labeled according to the PROTAC dose of ASA.

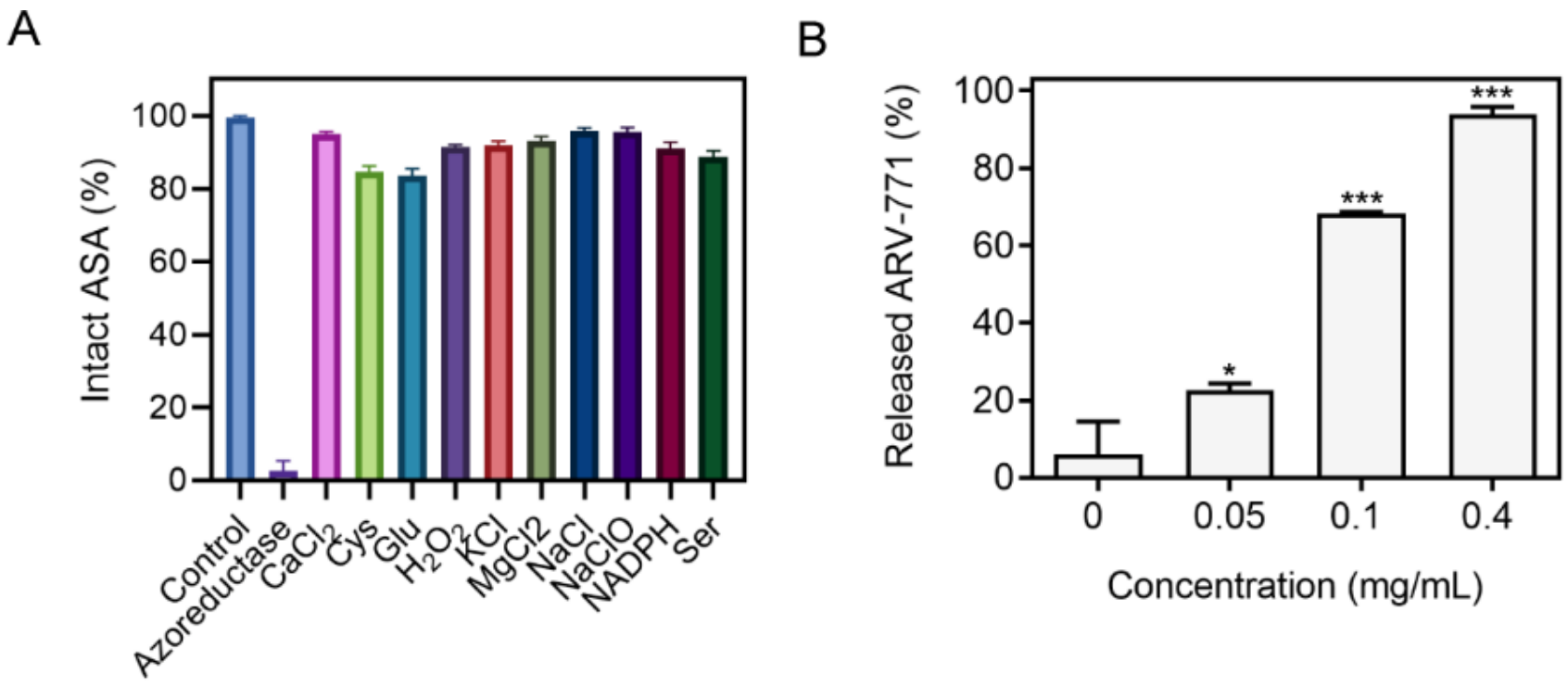

Figure S3 *In vitro* stability and ARV-771 release profile of ASA in the presence of diverse biological analytes. Related to Figure 1.

(A) Stability of ASA incubated with various biological analytes ($CaCl_2$, Cys, Glu, H_2O_2, KCl, $MgCl_2$, NaCl, NaClO, NADPH, Ser) under 20% O_2 for 24 h. Azoreductase-treated positive control group was incubated under 1% O_2 for 24 h.

(B) *In vitro* release rate of ARV-771 from ASA after 24-h incubation with a concentration gradient of azoreductase under 1% O_2.

Data are presented as mean ± SD. ns, not significant; *$p < 0.05$, **$p < 0.01$, ***$p < 0.001$ by one-way ANOVA.

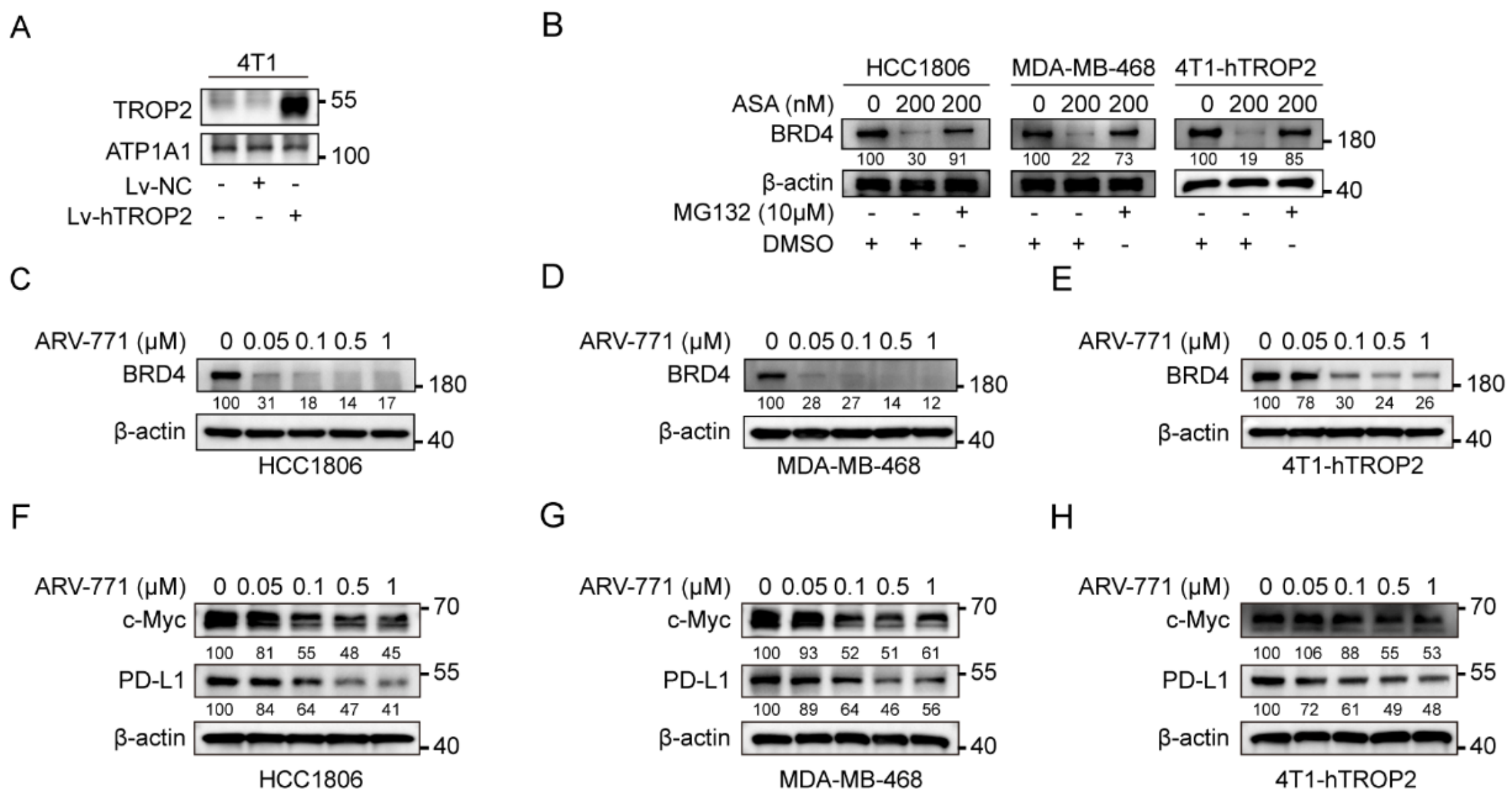

Figure S4 Validation of TROP2 overexpression, proteasome-dependent ASA activity, and ARV-771 action in TNBC cells. Related to Figures 1 and 2.

(A) Western blot analysis of TROP2 in 4T1 cells transduced with Lv-hTROP2 (lentiviral vector encoding human TACSTD2) or Lv-NC (empty vector).

(B) Western blot analysis of BRD4 in TNBC cells (HCC1806, MDA-MB-468, and 4T1-hTROP2) pretreated with DMSO (control) or MG132 (10 μM) for 1 h, followed by 6-h ASA treatment under 1% O_2.

(C-E) Western blot analysis of BRD4 in TNBC cells (HCC1806, MDA-MB-468, 4T1-hTROP2) treated with a concentration gradient of ARV-771 for 6 h under 1% O_2.

(F-H) Western blot analysis of c-Myc and PD-L1 in TNBC cells treated with a concentration gradient of ARV-771 for 24 h under 1% O_2.

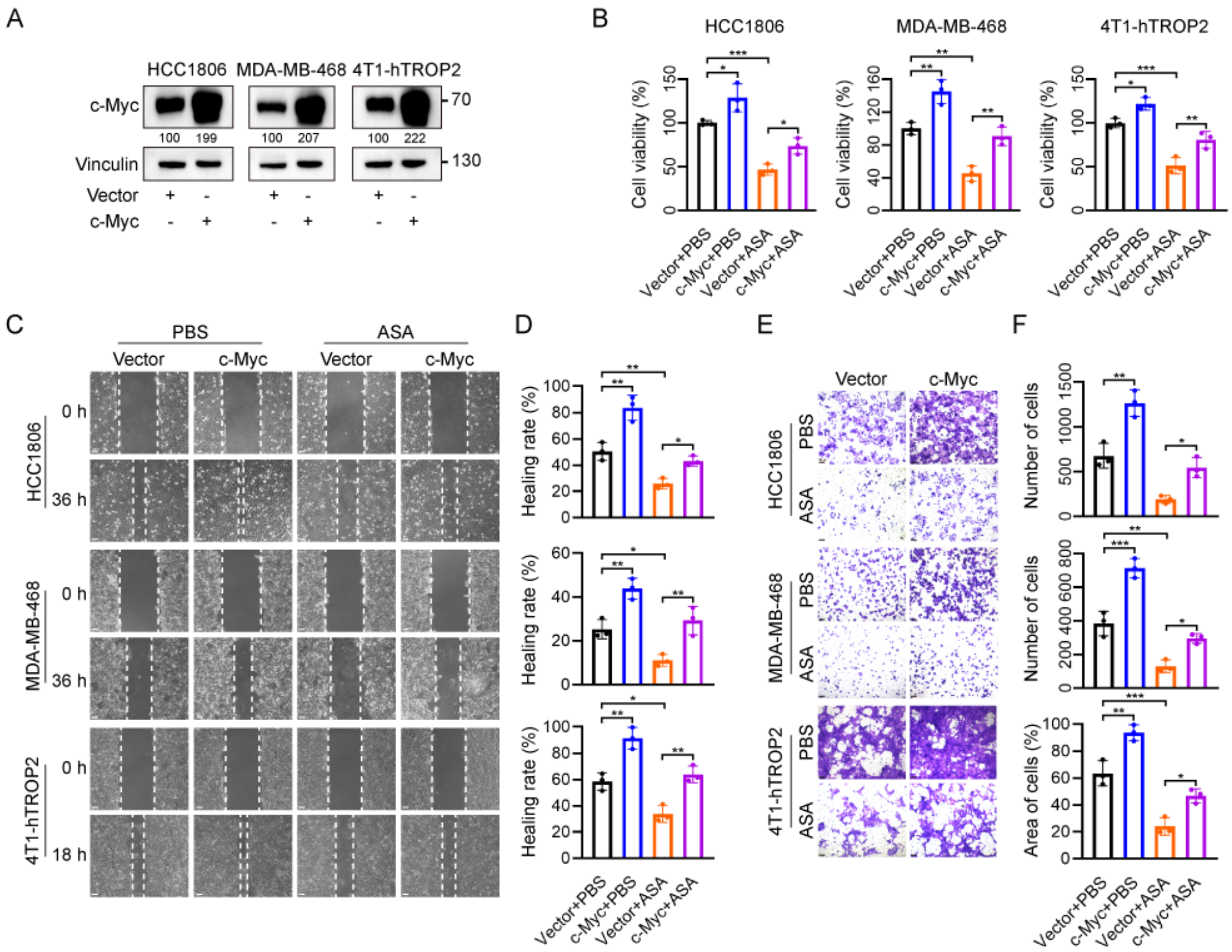

Figure S5 c-Myc overexpression rescues the antitumor efficacy of ASA. Related to Figure 2.

(A) Western blot analysis of c-Myc in TNBC cells (HCC1806, MDA-MB-468, 4T1-hTROP2) transfected with c-Myc overexpression plasmid or empty vector.

(B) Cell viability of c-Myc-overexpressing and vector control cells following 48-h treatment with ASA or PBS under 1% O_2.

(C and D) Migration of c-Myc-overexpressing and vector control cells following treatment with ASA or PBS under 1% O_2. Scale bars, 100 μm.

(E and F) Invasion of c-Myc-overexpressing and vector control cells following treatment with ASA or PBS under 1% O_2. Scale bars, 100 μm.

Data are presented as mean ± SD. ns, not significant; **p* < 0.05, ***p* < 0.01, ****p* < 0.001 by one-way ANOVA.

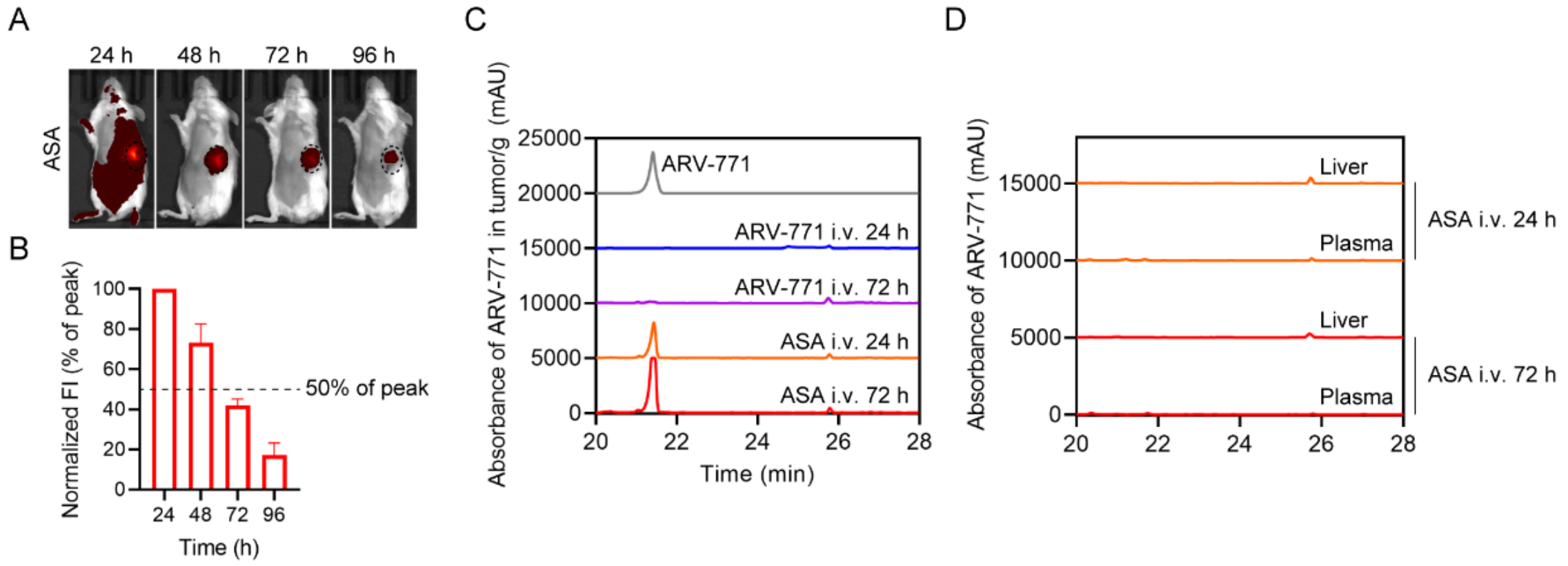

Figure S6 *In vivo* pharmacokinetic profile of ASA and tumor-specific ARV-771 release. Related to Figure 4.

(A and B**)** *In vivo* fluorescence imaging (A) and quantification (B) of normalized fluorescence intensity (FI) showing the biodistribution of Cy5-labeled ASA in 4T1-hTROP2-bearing mice post-administration (*n* = 4 mice per group).

(C) HPLC analysis of free ARV-771 levels in tumor tissues at 24 h and 72 h following treatment with ASA (10 mg/kg, i.v.) or free ARV-771 (0.32 mg/kg, i.v.) at equivalent PROTAC molar doses.

(D**)** HPLC analysis of free ARV-771 levels in liver and plasma at 24 h and 72 h after ASA administration. Data are presented as mean ± SD.

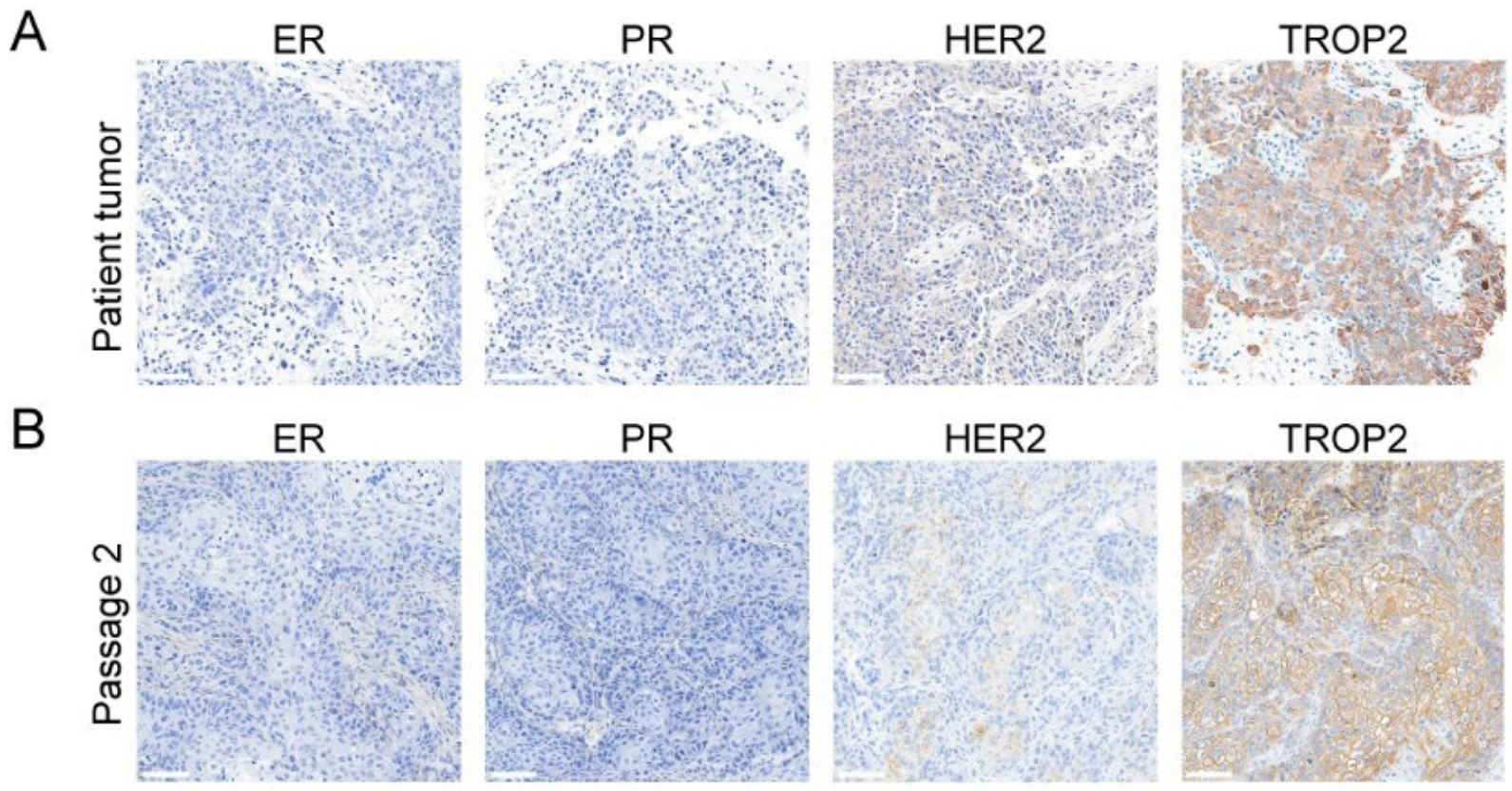

Figure S7 Histological characterization of the TNBC PDX model. Related to Figure 4.
(A and B) IHC staining for ER, PR, HER2, and TROP2 in the original patient tumor (A) and in the PDX tumor at passage 2 (B). Scale bar, 100 µm.

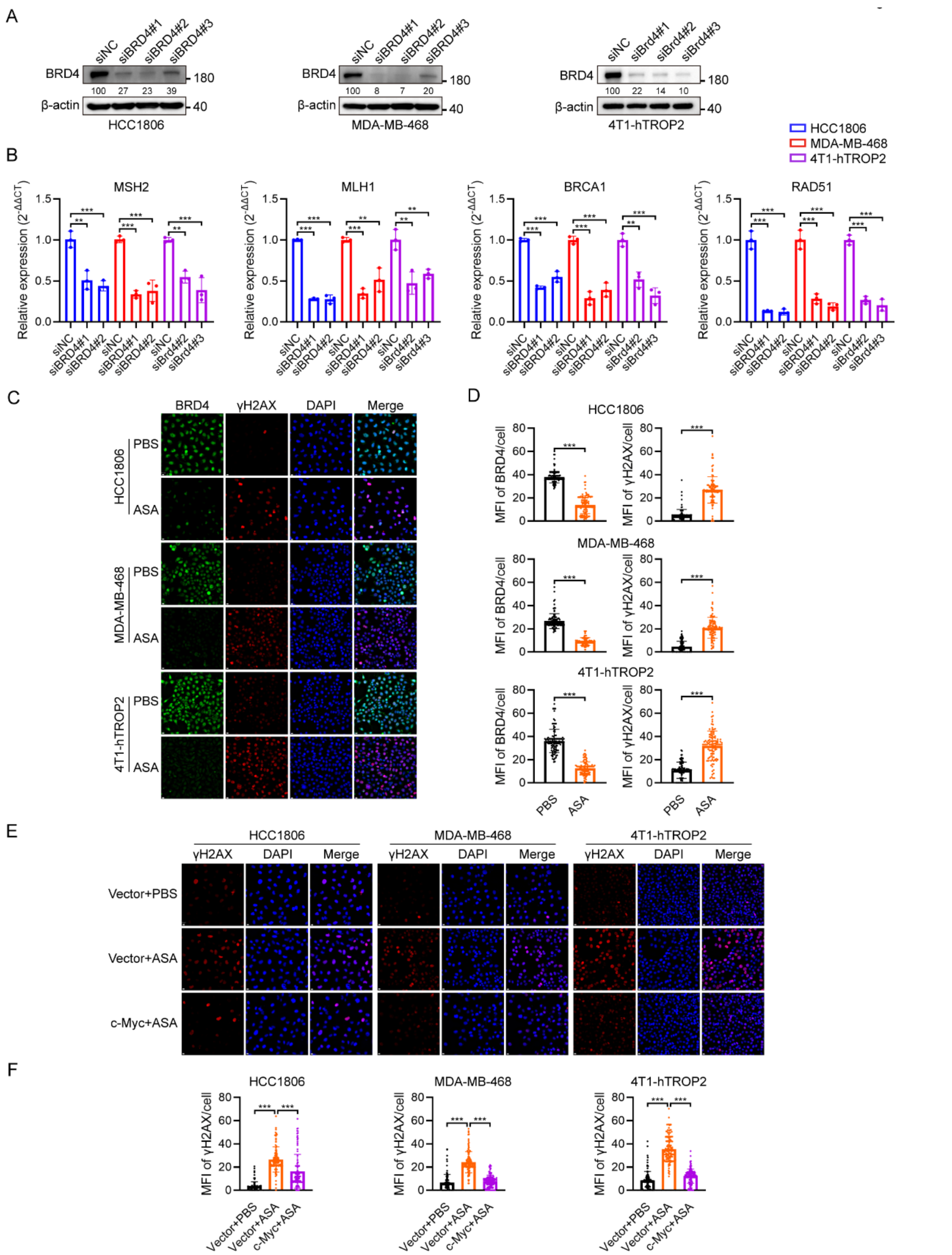

Figure S8 BRD4 knockdown impairs DNA repair gene expression and ASA treatment induces DNA damage in TNBC cells. Related to Figure 5.

(A) Western blot analysis of BRD4 in TNBC cells (HCC1806, MDA-MB-468, 4T1-hTROP2) following transfection with BRD4-targeting siRNA or control siRNA.

(B) qPCR analysis of MSH2, MLH1, BRCA1, and RAD52 in TNBC cells following transfection with BRD4-targeting siRNA or control siRNA.

(C and D) Representative immunofluorescence images (C) and quantification of per-cell MFI of BRD4 and γH2AX (D) in TNBC cells treated with ASA or PBS for

24 h under 1% O_2, followed by 2 h of camptothecin exposure, washout, and 4 h of recovery. Scale bars, 10 μm.
(E and F) Representative immunofluorescence images (E) and quantification of per-cell MFI of γH2AX (F) in c-Myc-overexpressing and vector control cells treated with ASA or PBS for 24 h under 1% O_2, followed by 2 h of camptothecin exposure, washout, and 4 h of recovery. Scale bars, 10 μm.
Data are presented as mean ± SD. ns, not significant; $^{*}p < 0.05$, $^{**}p < 0.01$, $^{***}p < 0.001$ by one-way ANOVA (B and F) or unpaired Student's t test (D).

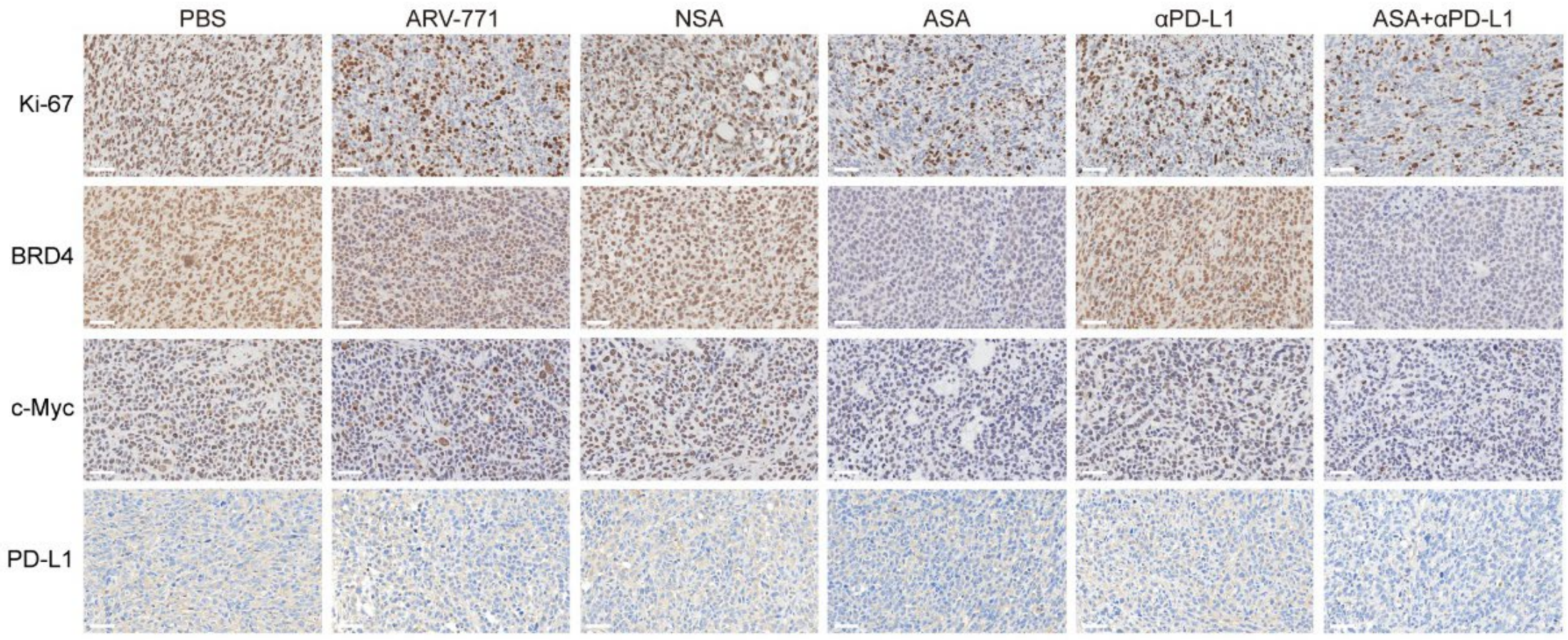

Figure S9 Representative IHC images of Ki-67, BRD4, c-Myc, and PD-L1 in tumor tissues of 4T1-hTROP2-bearing mice treated for 12 days with PBS, ARV-771 (5 mg/kg), NSA (10 mg/kg), ASA (10 mg/kg), αPD-L1 (5 mg/kg), or ASA plus αPD-L1. Scale bars, 100 μm. Related to Figure 5.

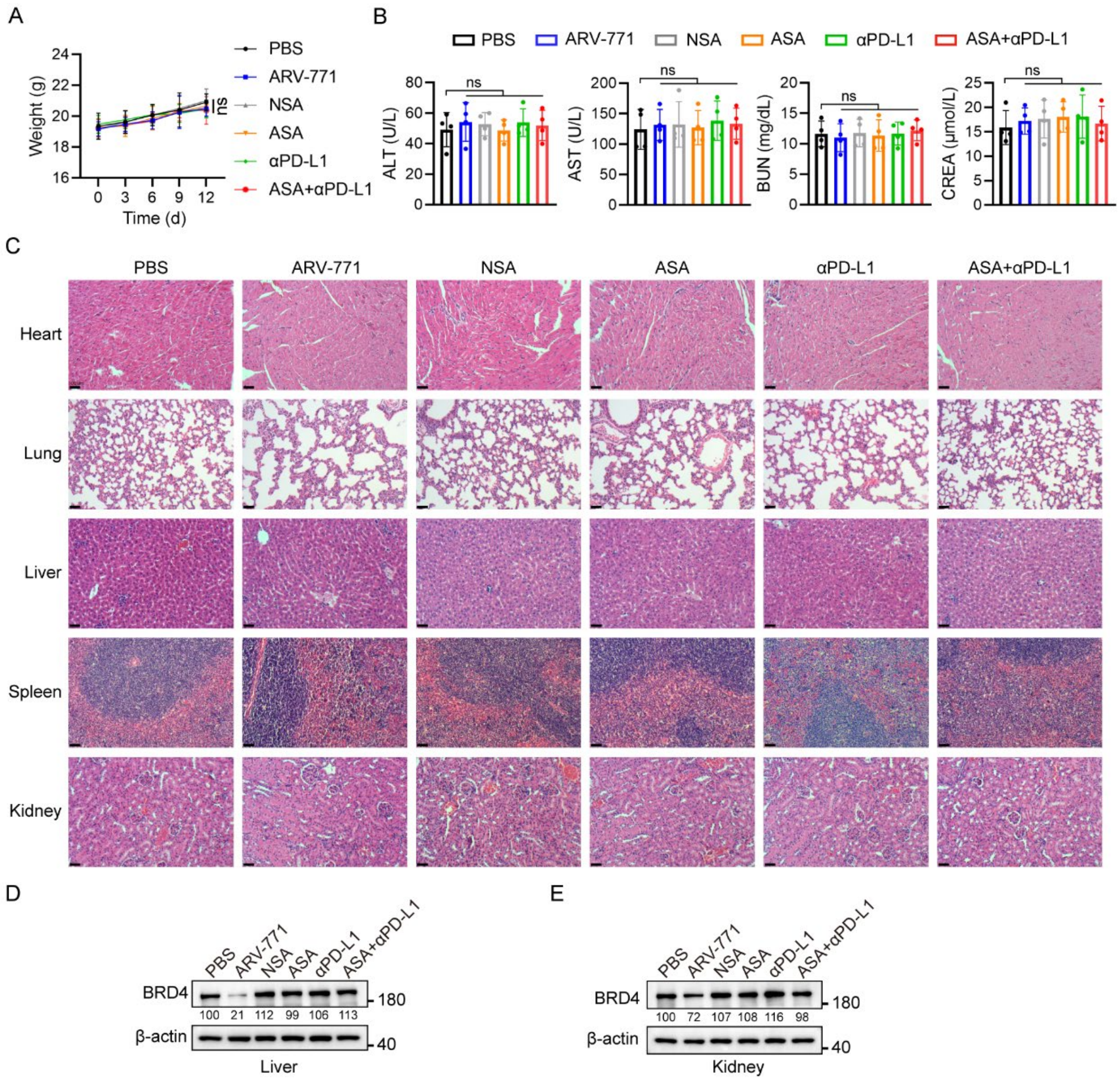

Figure S10 Biosafety profiling of different regimens. Related to Figure 5.

(A) Body weight changes of 4T1-hTROP2-bearing mice treated for 12 days with PBS, ARV-771 (5 mg/kg), NSA (10 mg/kg), ASA (10 mg/kg), αPD-L1 (5 mg/kg), or ASA plus αPD-L1.

(B and C) Serum levels of ALT, AST, BUN, and CREA (B) and representative H&E staining of major organs (heart, lung, liver, spleen, kidney) (C) from each treatment group. Scale bars, 100 μm.

(D and E) Western blot analysis of BRD4 in the liver (D) and kidney (E) of mice from each treatment group.

Data are presented as mean ± SD. ns, not significant; **p* < 0.05, ***p* < 0.01, ****p* < 0.001 by two-way ANOVA (A) or one-way ANOVA (B).

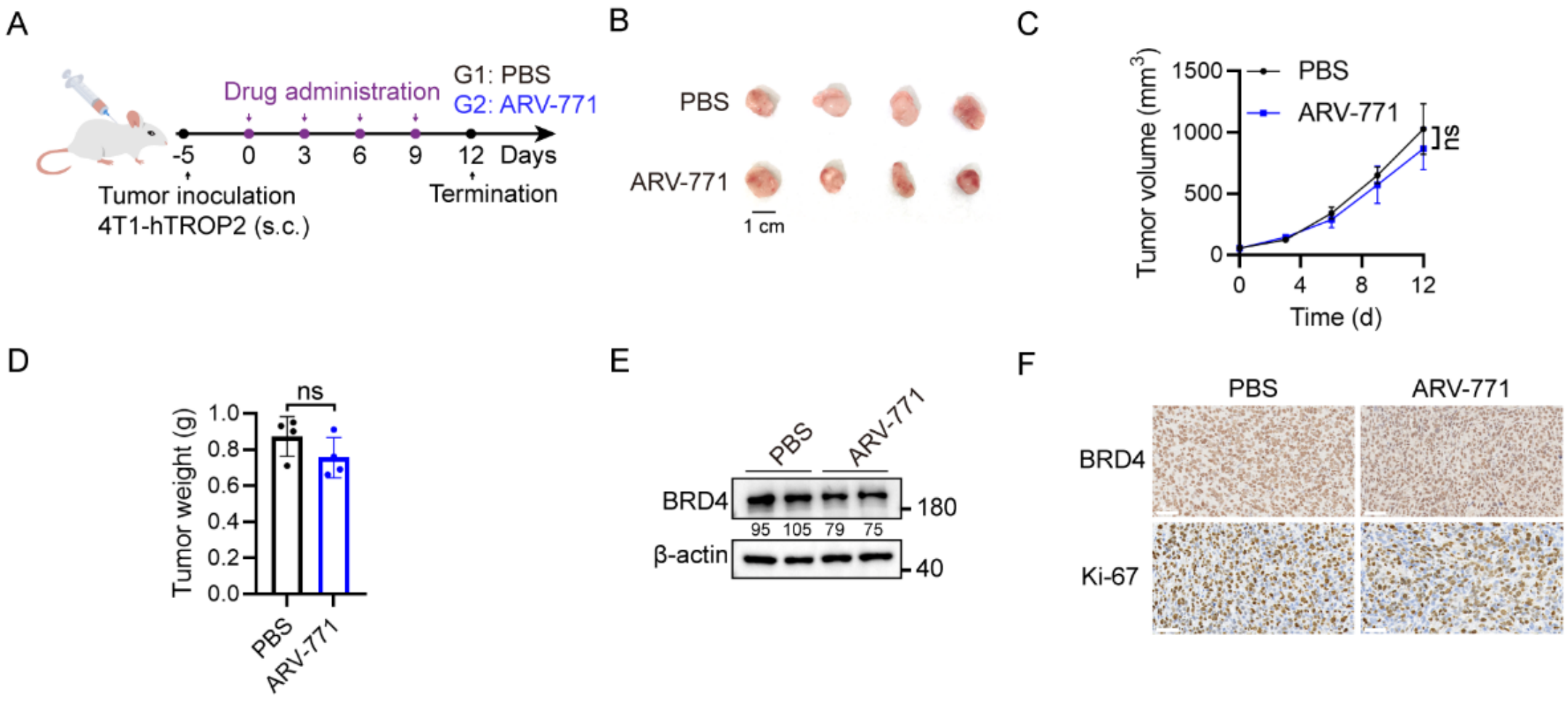

Figure S11 *In vivo* anti-tumor efficacy of ARV-771 at 0.32 mg/kg (equimolar to the PROTAC dose in 10 mg/kg ASA). Related to Figure 5.

(A) Schematic illustrating the treatment regimens. 4T1-hTROP2-bearing mice were treated for 12 days with PBS (i.v., q3d) or ARV-771 (0.32 mg/kg, i.v., q3d) (*n* = 4 mice per group).

(B-D) Tumor images (scale bar, 1cm) (B), tumor growth curves (C), and tumor weights (D).

(E) Western blot analysis of BRD4 in 4T1-hTROP2 tumor tissues from each treatment group.

(F) Representative IHC images of BRD4 and Ki-67 in 4T1-hTROP2 tumor tissues from each treatment group. Scale bars, 50 μm.

Data are presented as mean ± SD. ns, not significant; **p* < 0.05, ***p* < 0.01, ****p* < 0.001 by two-way ANOVA (C) or unpaired Student's t test (D).

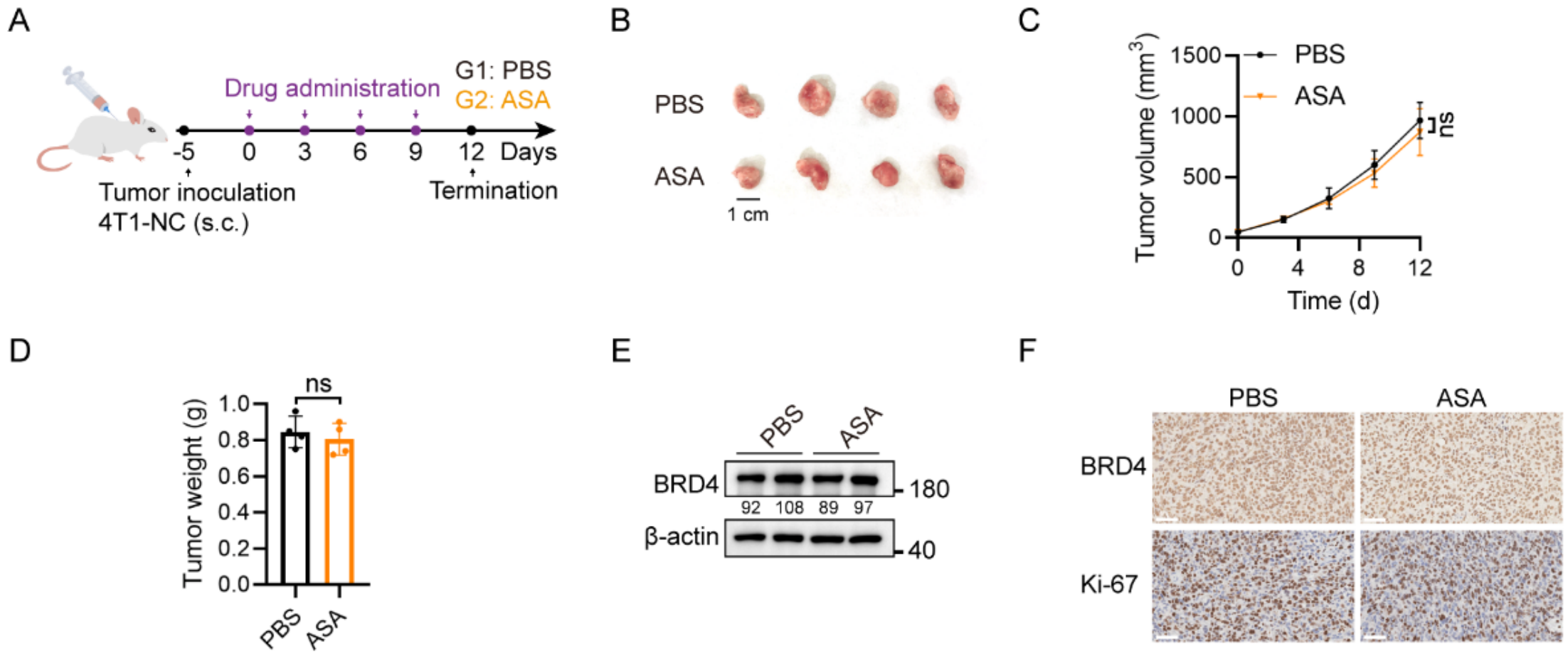

Figure S12 *In vivo* anti-tumor efficacy of ASA in 4T1-NC-bearing mice. Related to Figure 5.

(A) Schematic illustrating the treatment regimens. 4T1-NC-bearing mice were treated for 12 days with PBS (i.v., q3d) or ASA (10 mg/kg, i.v., q3d) (n=4 mice per group).

(B-D) Tumor images (scale bar, 1cm) (B), tumor growth curves (C), and tumor weights (D).

(E) Western blot analysis of BRD4 in 4T1-NC tumor tissues from each treatment group.

(F) Representative IHC images of BRD4 and Ki-67 in 4T1-NC tumor tissues from each treatment group. Scale bars, 50 μm.

Data are presented as mean ± SD. ns, not significant; *$p < 0.05$, **$p < 0.01$, ***$p < 0.001$ by two-way ANOVA (C) or unpaired Student's t test (D).

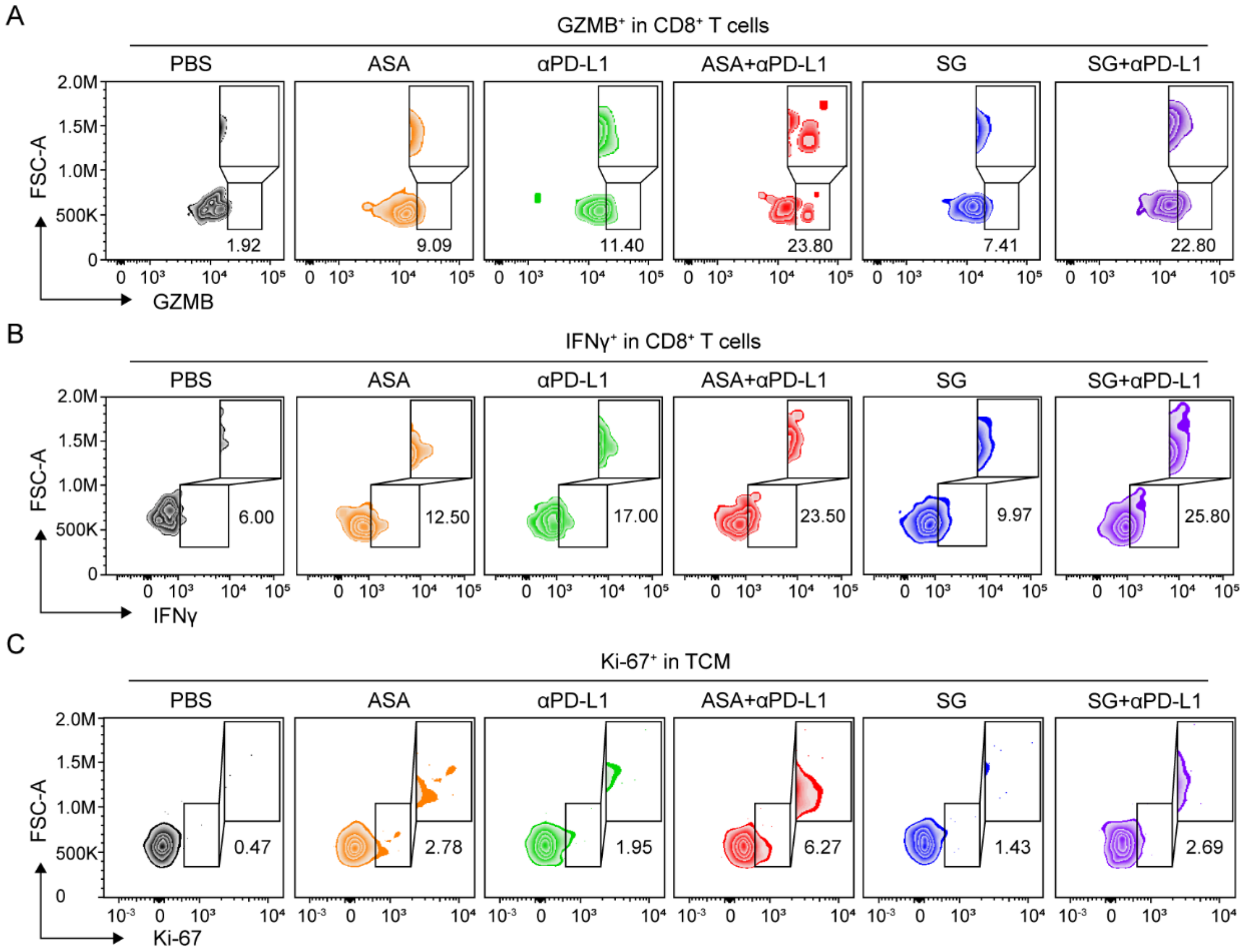

Figure S13 Representative plots of flow cytometry analysis. Related to Figures 6 and 7.

(A and B) Flow cytometry analysis of GZMB+ (A) and IFNγ+ (B) cell proportions within CD8+ T cells in lung metastatic tissue-infiltrating lymphocytes from mice treated with PBS, ASA (10 mg/kg), αPD-L1 (5 mg/kg), ASA (10 mg/kg) plus αPD-L1 (5 mg/kg), SG (10.27 mg/kg), or SG (10.27 mg/kg) plus αPD-L1 (5 mg/kg) for 12 days.

(C) Flow cytometry analysis of Ki-67+ cell proportions within the TCM population in splenic lymphocytes from mice in the long-term immunological memory evaluation assay.

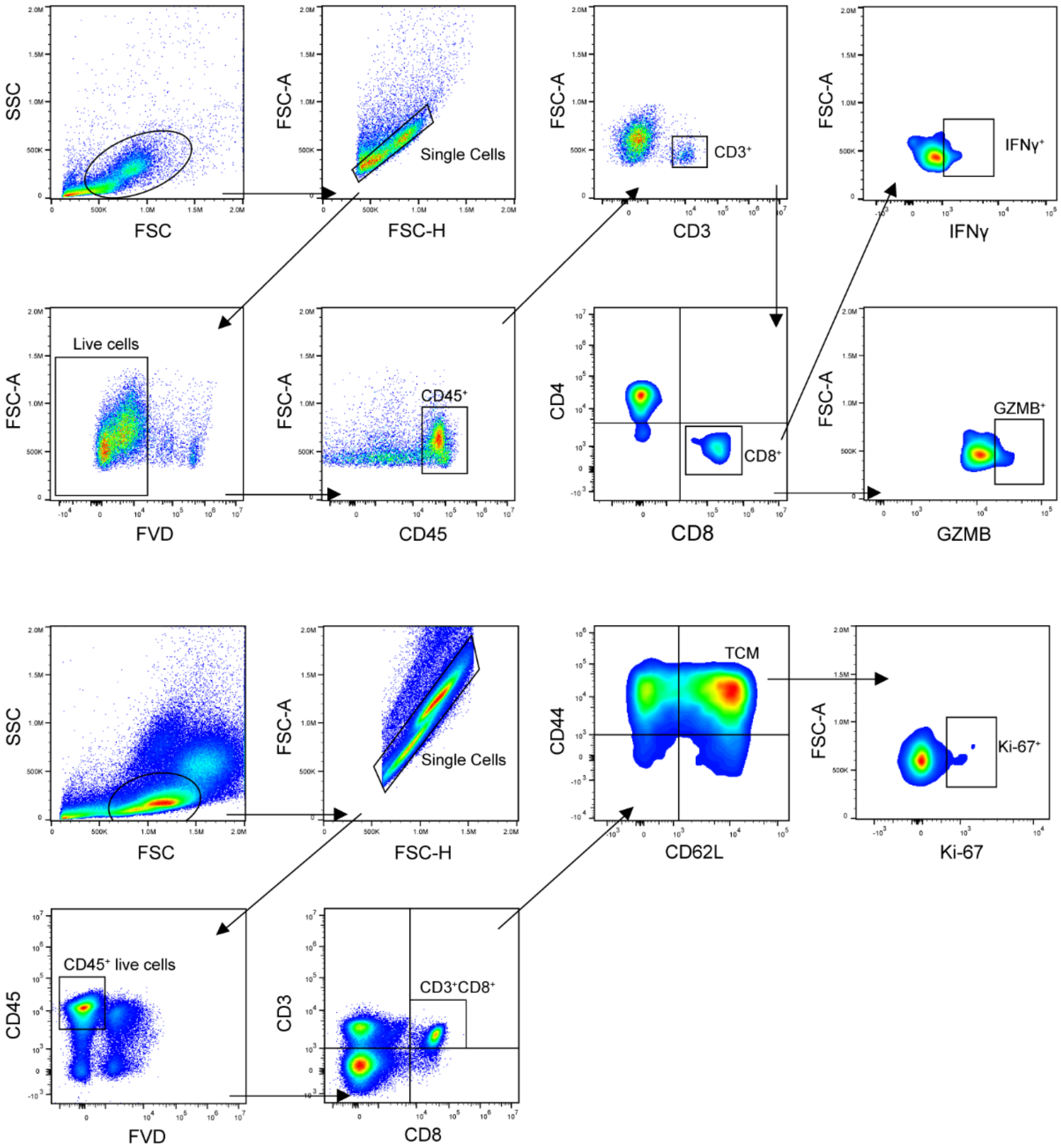

Figure S14 Gating strategies for flow cytometry analysis. Related to STAR Methods.

A

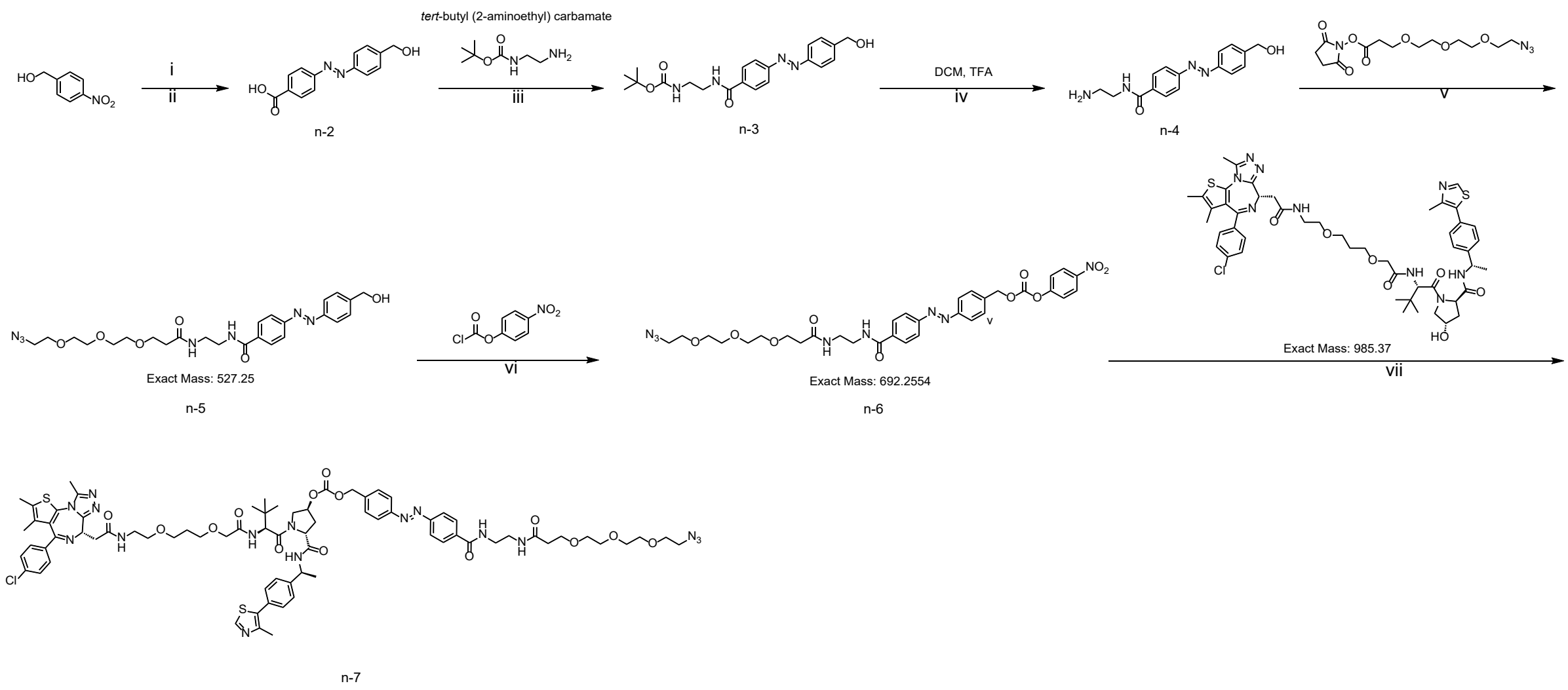

B

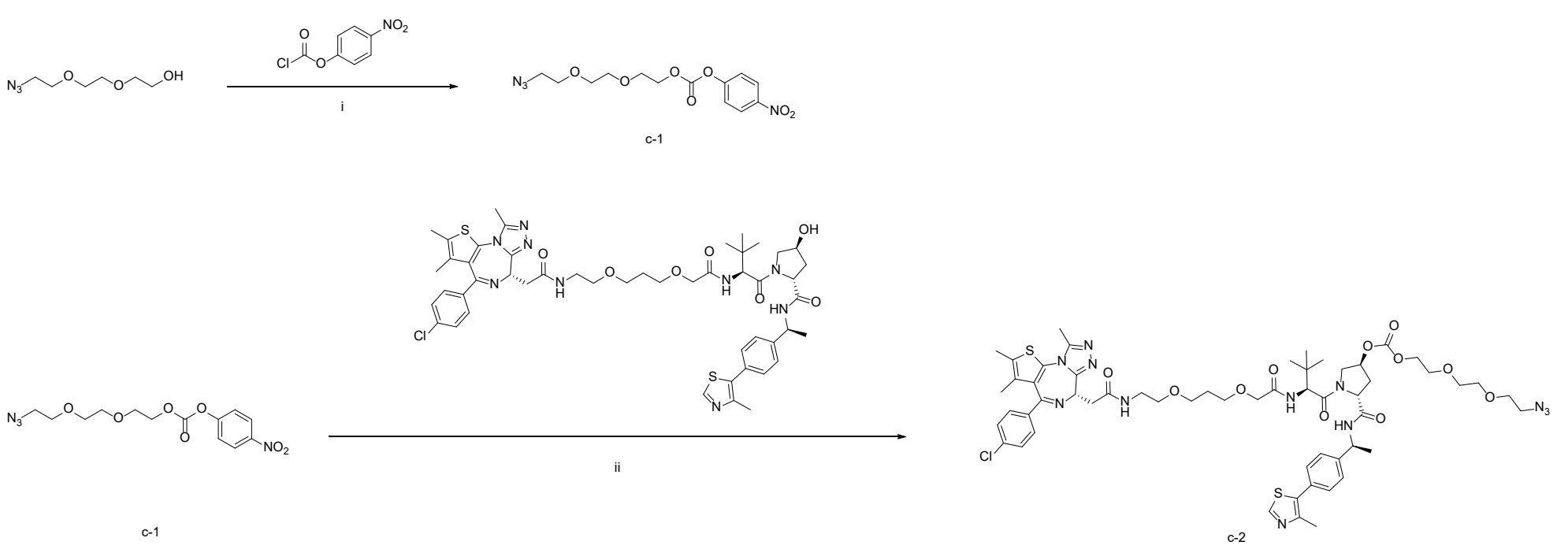

Figure S15 Synthetic routes of n-7 and c-2. Related to STAR Methods.

(A) Reagents and conditions. (i) 1. Zn, NH_4Cl, MeOH/H_2O, r.t., 30 min; 2. $FeCl_3$, MeOH/H_2O, -5 °C, 30 min; (ii) 4-aminobenzoic acid, AcOH, r.t., overnight; (iii) tert-butyl (2-aminoethyl) carbamate, EDCI, HOBt, DIPEA, DMF, r.t., overnight; (iv) trifluoroacetate, DCM, r.t., 6 h; (v) N_3-PEG$_3$-C$_2$-NHS ester, DIPEA, DMSO, r.t., overnight; (vi) 4-nitrophenyl carbonochloridate, DIPEA, DMAP, DCM, r.t., 5 h; (vii) ARV-771, Et_3N, DMAP, DCM, r.t., 72 h.

(B) Reagents and conditions. (i) 2-(2-(2-azidoethoxy) ethoxy) ethan-1-ol, 4-nitrophenyl carbonochloridate, Et_3N, DMAP, DCM, 0 °C to r.t., 4 h; (ii) ARV-771, Et_3N, DMAP, DCM, r.t., 72 h.

Name	ξmax ($M^{-1}cm^{-1}$) L=1 cm	Correction factors (Cfs)
Cy5	239000	0.06 cf1 0.04 cf2
Sacituzumab	218500	
IgG	204450	

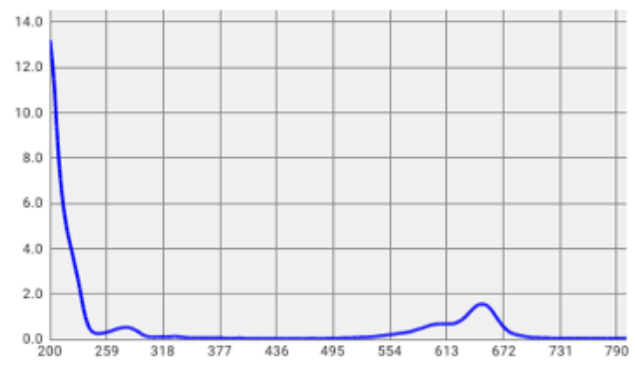

A650,Cy5=1.567
A280=0.539
[Cy5]=6.56 μM
[IgG]=2.18 μM

$$[Cy5]=\frac{A650,cy5}{\xi650,cy5} \quad A280=A_{280,antibody}+A_{280,cy5}$$

$$[antibody]=\frac{A_{280}-CF1*A_{650,cy5}}{\xi_{280,antibody}}$$

$$[X]=\frac{[Cy5]}{[antibody]}$$

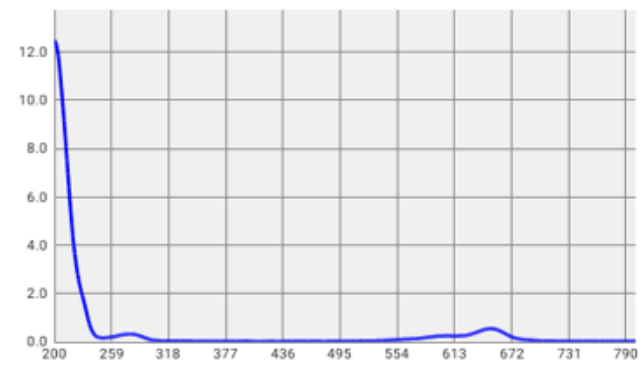

A650,Cy5=0.563
A280=0.336
[Cy5]=2.36 μM
[SAC]=1.38 μM

Figure S16 Characterization of Cy5-labeled antibodies. Related to STAR Methods. To measure Cy5-antibody ratio, Cy5-NHS was used to respectively tag Sacituzumab and IgG, whose amounts were further quantified by measuring Cy5 according to the Lambert-Beer law. UV-vis absorbance spectra of Cy5-labeled Sacituzumab and IgG. The Cy5-to-antibody ratio (X) and was calculated using formula described in the figure. X was measured after Sacituzumab and IgG were labeled with Cy5 and further used to determine Sacituzumab and IgG concentration.

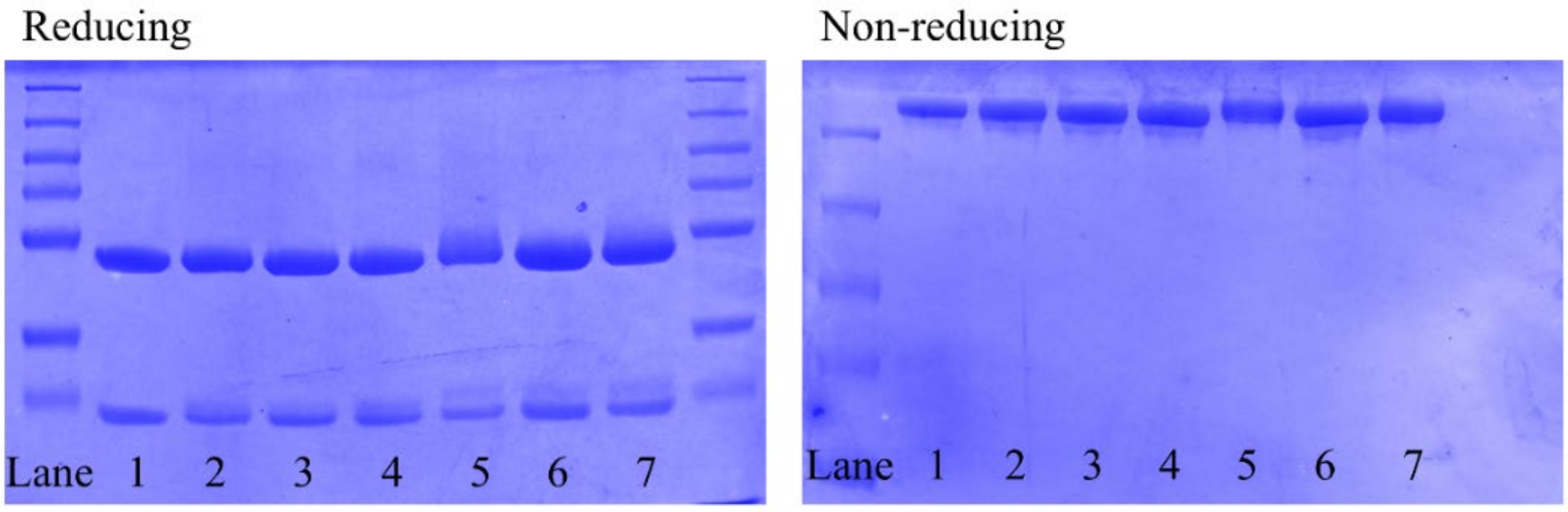

Figure S17 Coomassie-stained SDS-PAGE of Sacituzumab and ASA variants under reducing and non-reducing conditions. Related to STAR Methods. Lane 1: Sacituzumab; Lane 2: Sacituzumab-2DBCO; Lane 3: Sacituzumab-5DBCO; Lane 4: Sacituzumab-8DBCO; Lane 5-7: ASA with DAR=2 (Lane 5), DAR=5 (Lane 6), DAR=8 (Lane 7).

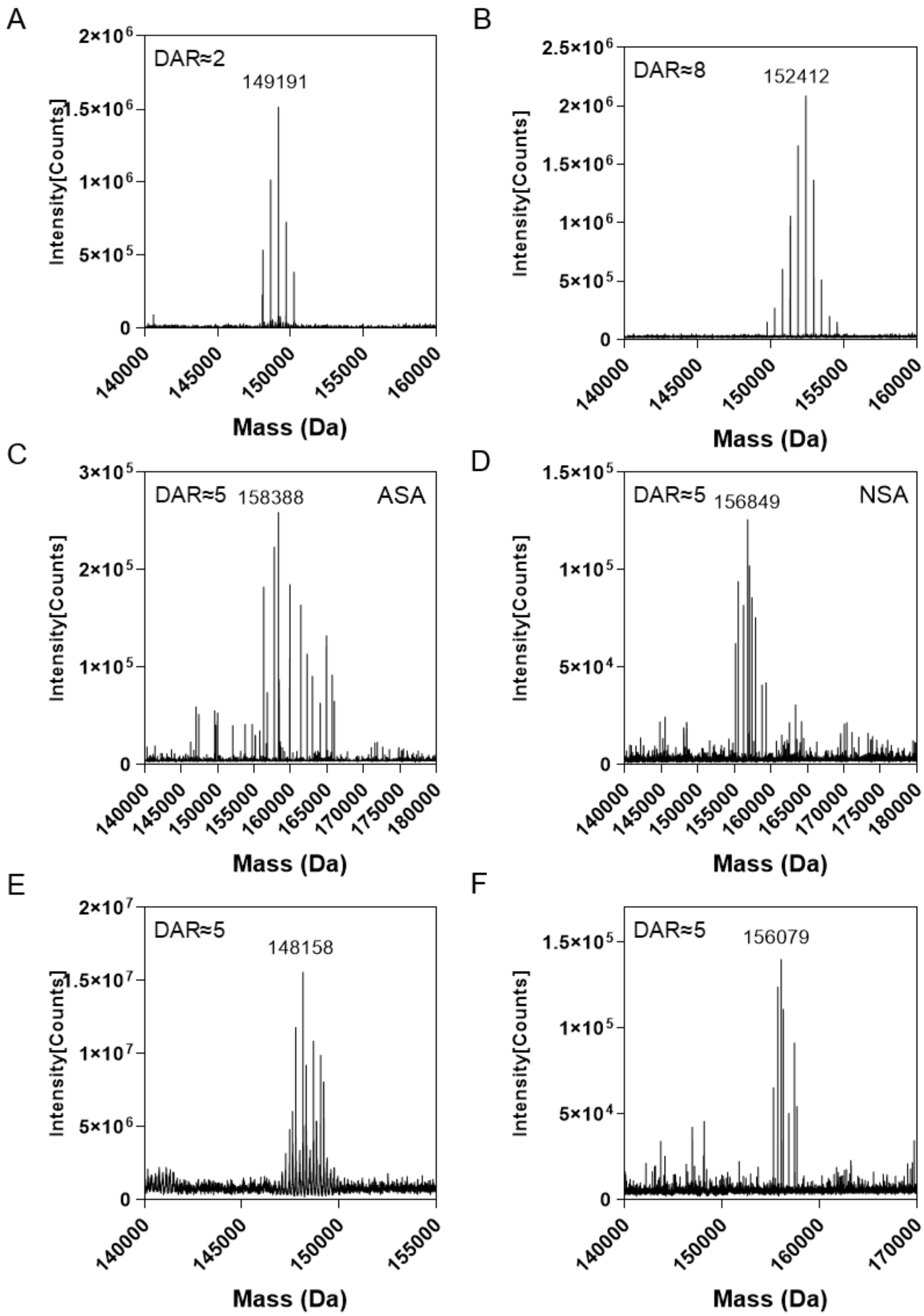

Figure S18 Intact mass spectrometry analysis of antibody-PROTAC conjugates and related controls. Related to STAR Methods.

(A-D) Intact mass spectrometry analysis of ASA variants (A-C) and NSA (D). To obtain the payload for antibody conjugation, n-6 (13 mg, 0.019 mmol) and ARV-771 (25 mg, 0.025 mmol) were dissolved in anhydrous dichloromethane (10 mL) under nitrogen protection, and the mixture was treated with DMAP (catalytic amount) and TEA in an ice bath, then stirred at room temperature for 3 days. The mixture was purified by HPLC to obtain the desired product n-7 (18 mg, 52% yield), whose structure was confirmed by ^{1}H NMR ($CDCl_3$, 400 MHz): δ = 7.36 (d, *J* = 9.0 Hz, 1H), 6.74 (dd, *J* = 9.0, 2.6 Hz, 1H), 6.55 (d, *J* = 2.6 Hz, 1H), 6.21 (s, 1H), 5.22 (s, 2H),

4.04 (s, 2H), 3.58 (t, *J* = 7.6 Hz, 2H), 3.11 (t, *J* = 7.8 Hz, 2H), 2.39 (s, 3H), 2.19 (s, 3H), 1.47 (s, 9H); ^{13}C NMR ($CDCl_3$, 100 MHz): 195.72, 170.20, 168.84, 161.56, 155.88, 150.59, 149.28, 124.64, 109.09, 107.99, 98.85, 82.64, 61.27, 53.83, 52. 08, 30.72, 28.07, 26.21, 20.74. Calc'd $[M+H]^+$=1539.6111; found 1539.6191. This payload was subsequently conjugated to DBCO-modified Sacituzumab (DAR ≈ 2, 5, and 8) to generate ASA variants, while NSA was obtained by conjugating DBCO-SAC (DAR ≈ 5) with c-2.

(E and F) Intact mass spectrometry analysis of DBCO-IgG (E) and IgG-ARV-771 (F). IgG-ARV-771 was generated by conjugating n-7 to DBCO-modified IgG under the same conditions as ASA.

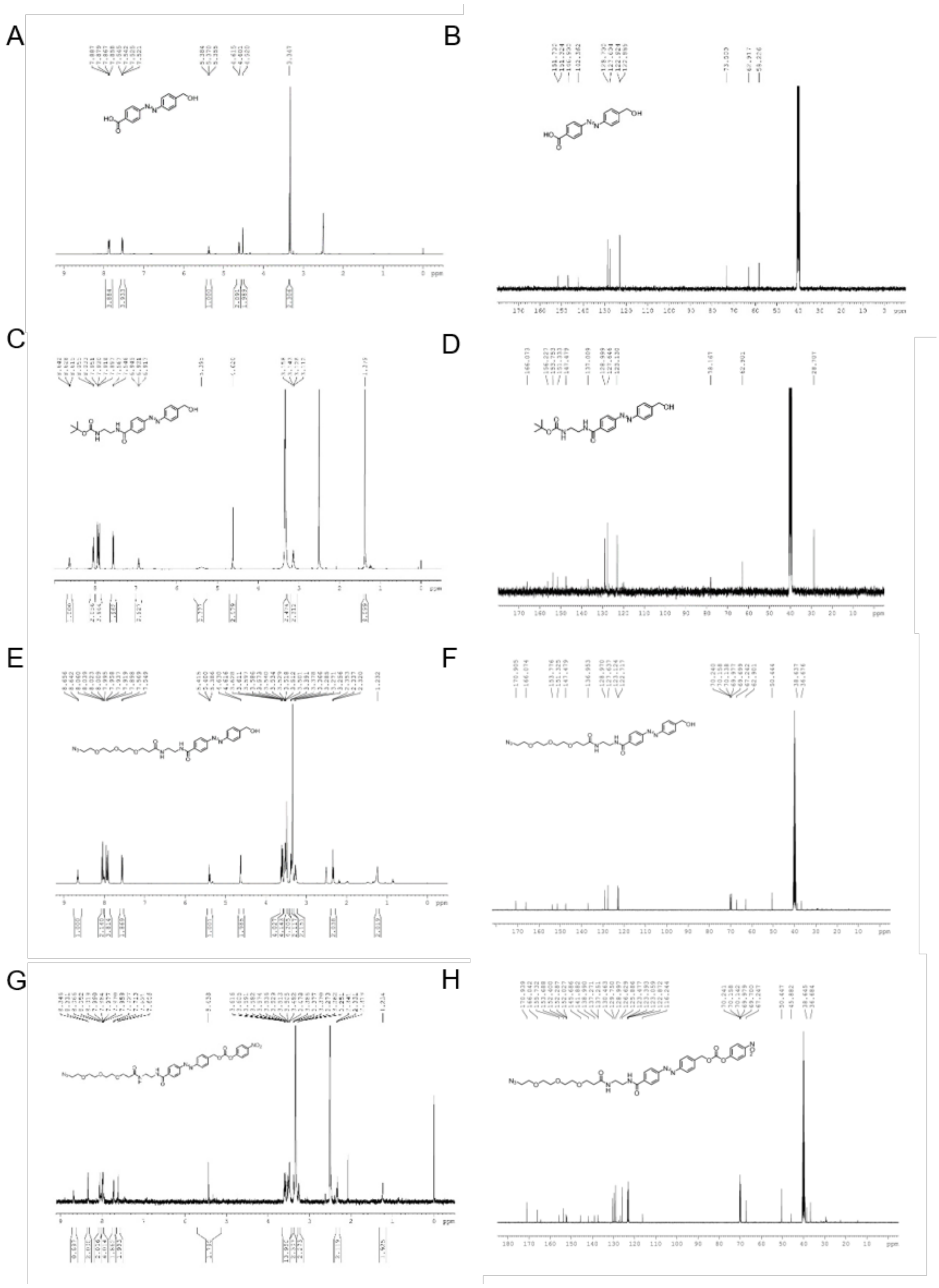

Figure S19 ^{1}H NMR and ^{13}C NMR spectra of compounds n-2 to n-6. Related to STAR Methods.

(A and B) ^{1}H NMR and ^{13}C NMR of n-2. p-Nitrobenzyl alcohol (6.0 g, 39.18 mmol) was dissolved in methanol (60 mL), then zinc powder (7.69 g, 117.54 mmol) was added, and followed by the slow dropwise addition of the aqueous solution of NH_4Cl (3.14 g, 58.77 mmol; 15 mL) with stirring. After stirring the reaction for 30 min at room temperature, the insoluble solids were removed by filtration, and the resulting filtrate was cooled in an ice-salt bath. Subsequently, $FeCl_3$ (58.77 mmol) dissolved in methanol/water (30 mL/15 mL) was slowly added dropwise to the cooled filtrate.

After completion of the dropwise addition, the mixture was continued to be stirred at low temperature for 30 min. The insolubles in the mixture were removed by filtration again. Saturated brine was added to the filtrate, followed by extraction with DCM. The organic phases were combined, washed with saturated brine three times, dried over sodium sulfate, and concentrated under reduced pressure to obtain an orange-yellow solid. The above solid was directly dissolved in acetic acid (50 mL), followed by the addition of p-aminobenzoic acid (2.7 g, 19.69 mmol), and the mixture was stirred at room temperature overnight. After completion of the reaction, the insoluble solid precipitated during the reaction was separated by filtration. The filter cake was washed with DCM to obtain an orange solid. Finally, further purification was performed by silica column chromatography to give n-2 as a final product (2.1 g, 41 % yield). ^{1}H NMR (400 MHz, DMSO-*d6*): δ = 7.87 (dd, *J* = 8.32, 3.28 Hz, 4H), 7.53 (dd, *J* = 8.4 Hz, 4H), 5.37 (t, *J* = 5.72, 1H), 4.61 (d, *J* = 5.6 Hz, 2H), 4.52 (s, 2H), 3.35 (s, 2H). ^{13}C NMR (100 MHz, DMSO-*d6*): 151.73, 151.32, 146.90, 142.36, 128.70, 127.60, 122.92, 122.89, 73.50, 62.92, 58.23.

(C and D) ^{1}H NMR and ^{13}C NMR of n-3. The compound n-2 (2.1 g, 8.2 mmol) was dissolved in DMF (20 mL), then tert-butyl (2-aminoethyl) carbamate (1.7 g, 10.7 mmol), EDCI (1.9 g, 9.9 mmol), and HOBt (1.3 g, 9.9 mmol) DIPEA (3.2 g, 24.6 mmol) and catalytic amount of DMAP were added, and the mixture was stirred at room temperature overnight. After completion of the reaction, the solvent was concentrated under reduced pressure. Finally, further purification was performed by silica column chromatography to obtain n-3 as an orange-yellow solid (2.55 g, 78% yield). ^{1}H NMR (400 MHz, DMSO-*d6*): δ = 8.63 (t, *J* = 5.4 Hz, 1H), 8.04 (d, *J* = 8.4 Hz, 2H), 7.95-7.90 (m, 4H), 7.55 (d, *J* = 8.4 Hz, 2H), 6.93 (t, *J* = 5.6 Hz, 1H), 5.40 (br, 1H), 4.62 (s, 2H), 3.31 (m, 2H), 3.14 (q, 2H), 1.38 (s, 9H). ^{13}C NMR (100 MHz, DMSO-*d6*): 166.07, 156.22, 153.75, 151.33, 147.48, 137.01, 129.00, 127.65, 123.13, 78.17, 62.90, 28.71.

(E and F) ^{1}H NMR and ^{13}C NMR of n-5. To a solution of n-3 (2.55 g, 6.4 mmol) in DCM (20 mL), TFA (10 mL) was added, and the mixture solution was stirred 6 hours at room temperature. The solution was concentrated under reduced pressure and vacuum pump for 4 hours. After completion of the reaction, the solvent was concentrated under reduced pressure, the solid was used for next step without further purification. To a solution of n-4 (1.8 g, 6 mmol) in DMSO (10 mL), N_3-PEG$_3$-C$_2$-NHS ester and DIPEA was added, and the mixture solution was stirred 12 hours at room temperature. The mixture was purified by HPLC to obtain the desired product n-5 (2.1 g, 65 % yield). ^{1}HNMR (400 MHz, DMSO-*d6*): δ = 8.66 (t, *J* = 5.4 Hz, 1H), 8.10-7.89 (m, 6H), 7.56 (d, *J* =8.3, 2H), 5.40 (t, *J* = 11.4 Hz, 1H), 4.62 (d, *J* = 5.6 Hz, 2H), 3.63-3.57 (m, 4H), 3.54-3.50 (m, 4H), 3.48 (s, 4H), 3.38 (t, *J* = 5.1 Hz, 2H), 3.27 (t, *J* = 5.7 Hz, 2H), 2.34 (t, *J* = 6.5 Hz, 2H), 1.23 (s, 2H); ^{13}C NMR (100 MHz, DMSO-*d6*): 170.90, 166.07, 153.78, 153.69, 151.33, 147.48, 136.95, 128.97, 127.64, 123.12, 122.72, 70.24, 70.16, 70.14, 69.98, 69.70, 67.24, 62.90, 50.44, 38.64, 36.68.

(G and H) ^{1}H NMR and ^{13}C NMR of n-6. To a solution of n-5 (2.1 g, 4 mmol) in 20 mL DCM, 4-nitrophenyl carbonochloridate (2.4 g, 12 mmol), 1.5 eq Et_3N and catalytic amount of DMAP were added under 0℃, then the mixture was stirred at room temperature for 4 h. After completion of the reaction monitored by TLC, the mixture was concentrated under reduced pressure. The mixture was purified by

HPLC to obtain the desired product n-6 (1.3 g, 48% yield). ^{1}HNMR (400 MHz, DMSO-*d6*): δ = 8.68 (s, 1H), 8.34 (d, *J* = 6.0 Hz, 2H), 8.05 (d, *J* = 5.6, 4H), 7.98 (m, 4H), 7.72 (d, *J* = 5.6 Hz, 2H), 7.61 (d, *J* = 6.0 Hz, 2H), 6.44 (s, 1H), 3.62-3.48 (m, 14H), 3.38 (t, *J* = 3.6 Hz, 2H), 3.26 (t, *J* = 4.4 Hz, 2H), 2.33 (t, *J* = 4.0 Hz, 2H), 1.23 (s, 2H); ^{13}C NMR (100 MHz, DMSO-*d6*): 170.94, 166.04, 155.73, 153.69, 152.40, 152.29, 152.03, 145.69, 141.88, 138.99, 137.27, 137.25, 130.46, 129.75, 129.00, 126.63, 125.89, 123.48, 123.39, 123.06, 122.87, 116.24, 70.24, 70.16, 70.14, 69.98, 69.70, 67.25, 50.45, 45.88, 38.65, 36.68.

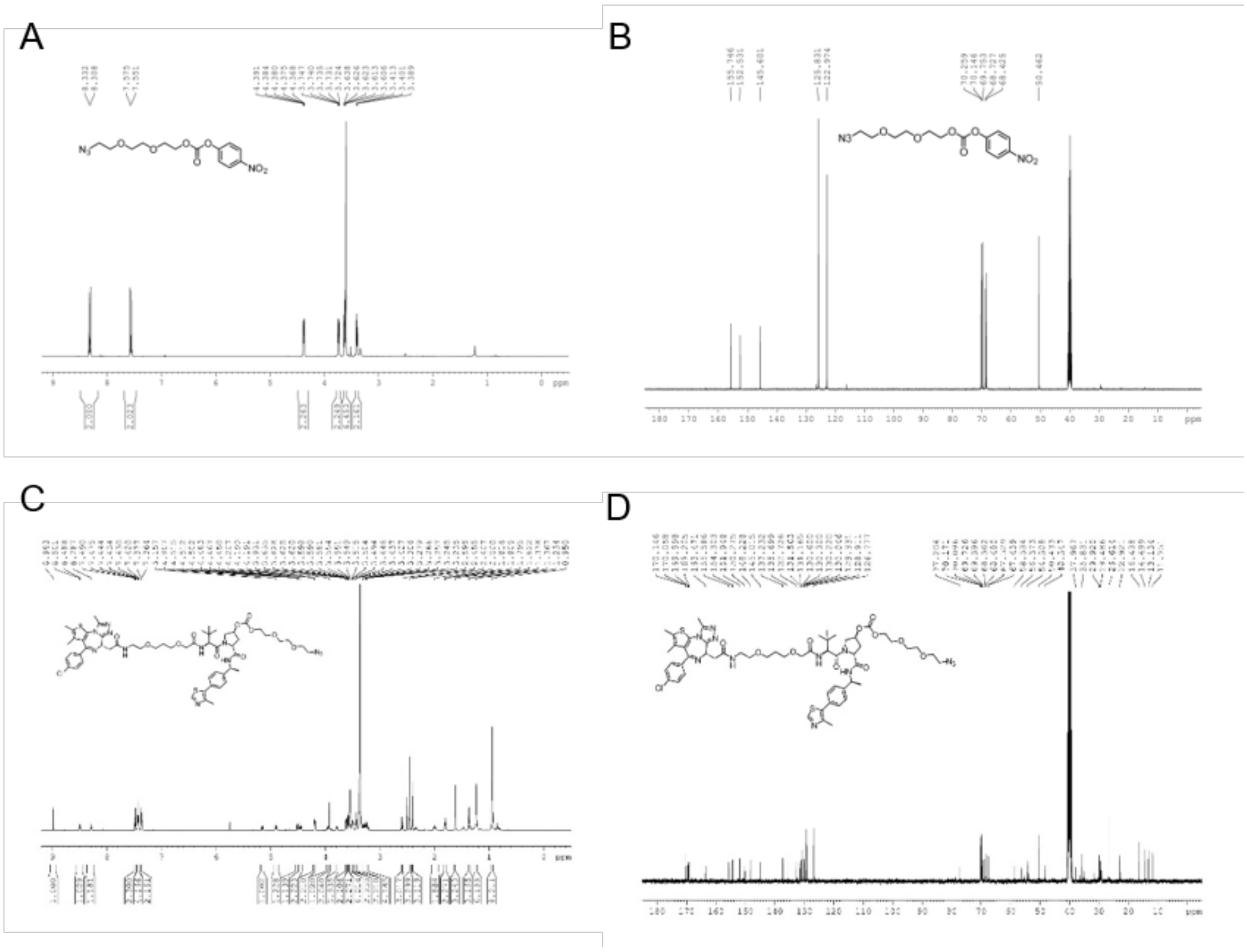

Figure S20 ^{1}H NMR and ^{13}C NMR spectra of compounds c-1 and c-2. Related to STAR Methods.

(A and B) ^{1}H NMR and ^{13}C NMR of c-1. To a solution of 2-(2-(2-azidoethoxy) ethoxy) ethan-1-ol (35 mg, 0.2 mmol) in 5 mL DCM, 4-nitrophenyl carbonochloridate (24 mg, 0.6 mmol), 1.5 eq Et_3N and catalytic amount of DMAP were added under 0℃, then the mixture was stirred at room temperature for 4 h. After completion of the reaction monitored by TLC, the mixture was concentrated under reduced pressure. Finally, further purification was performed by silica column chromatography to obtain c-1 as a yellow oil (54 mg, 80% yield). $^{1}HNMR$ (400 MHz, DMSO-*d6*): δ = 8.32 (d, *J* = 9.6 Hz, 2H), 7.56 (d, *J* = 9.6 Hz, 2H), 4.38 (m, 2H), 3.74 (m, 2H), 3.64-3.60 (m, 6H), 3.40 (t, *J* = 5.1 Hz, 2H); ^{13}C NMR (100 MHz, DMSO-*d6*): 155.75, 152.53, 145.60, 125.83, 122.97, 70.26, 70.15, 69.75, 68.73, 68.43, 50.46.

(C and D) ^{1}H NMR and ^{13}C NMR of c-2. Under nitrogen protection, a mixture of c-1 (10 mg, 0.03 mmol) and ARV-771 (20 mg, 0.02 mmol) in anhydrous dichloromethane (10 mL) was added with DMAP (catalytic amount) and TEA in an ice bath, and stirred at room temperature for 3 days. The mixture was purified by HPLC to obtain the desired product c-2 (12 mg, 52% yield). ^{1}H NMR (600 MHz, DMSO-*d6*): δ = 8.98 (s, 1H), 8.49 (d, 7.6 Hz, 1H), 8.29 (t, *J* = 5.6 Hz, 1H), 7.48 (m, 2H), 7.43 (m, 4H), 7.37 (m, 2H), 5.16 (s, 1H), 4.91 (m, 1H), 4.53 – 4.44 (m, 3H), 4.20 (m, 2H), 3.96 (d, *J* = 12.0, 3.6, 1H), 3.93 (s, 2H), 3.80 -3.78 (dd, *J* = 12.0, 2.6 Hz, 2H), 3.64-3.62 (m, 2H), 3.59 (t, *J* = 4.8 Hz, 2H), 3.56 -3.53 (m, 6H), 3.50 (t, *J* = 6.1 Hz, 2H), 3.44 (t, *J* = 5.8 Hz, 2H), 3.33 -3.20 (m, 4H), 2.60 (s, 3H), 2.46 (s, 3H), 2.41 (s, 3H), 2.02 -1.96 (m, 2H), 1.84 -1.77 (m, 2H), 1.37 (d, *J* = 7.0 Hz, 3H), 1.23 (s, 6H), 0.95 (s, 9H); ^{13}C NMR ($CDCl_3$, 100 MHz): 170.15, 170.06, 169.60, 169.21, 163.47, 155.88, 155.59, 154.30, 151.95, 150.28, 148.23, 145.01, 137.23, 135.70,

132.73, 131.56, 131.17, 130.60, 130.30, 130.22, 130.05, 129.33, 128.91, 126.78, 77.20, 70.17, 70.10, 69.73, 69.40, 68.56, 68.48, 67.53, 67.44, 58.53, 56.37, 54.31, 50.43, 48.35, 37.97, 35.83, 29.92, 29.49, 26.61, 22.92, 16.44, 14.50, 13.13, 11.75. Calc'd $[M+Na]^+$=1209.4395; found 1209.4355.

Table S1 DC_{50} values (nM) of BRD4 degradation by the indicated compounds in TNBC cells. Related to Figures 2 and S4.

Compound	HCC1806	MDA-MB-468	4T1-hTROP2
ARV-771	3.6 ± 0.9	6.1 ± 0.7	76.0 ± 12.1
ASA	50.0 ± 5.1	102.4 ± 8.5	96.2 ± 12.7

Data represent mean ± SEM from three independent experiments.

Table S2 IC_{50} values (nM) of the ASA in TNBC cells. Related to Figure 2.

Compound	HCC1806	MDA-MB-468	4T1-hTROP2
ASA	82.3 ± 12.8	141.5 ± 17.4	117.4 ± 12.4

Data represent mean ± SEM from three independent experiments.

Table S3 siRNA sequences used for BRD4 knockdown in TNBC cells. Related to STAR Methods.

siRNA	Species	Sequence (5′→3′)
siBRD4 #1	*Homo sapiens*	CCGUGAUGCUCAGGAGUUU
siBRD4 #2	*Homo sapiens*	GCACAAUCAAGUCUAAACUGG
siBRD4 #3	*Homo sapiens*	AAGACAAAGAAGGGAGUG
siBrd4 #1	*Mus musculus*	GCACAAUCAAGUCUAAACUAG
siBrd4 #2	*Mus musculus*	CAAUCAAGUCUAAACUAGAGU
siBrd4 #3	*Mus musculus*	GUGUAGUACACAUAAUUCAGU
siNC[a]	-	UUCUCCGAACGUGUCACGU

[a] siNC, a non-targeting scramble sequence

Table S4 The primers for qRT-PCR. Related to STAR Methods.

Genes	sense	Antisense
MSH2	CAGCAGTCAGAGCCCTTAACCT	GAGAGGCTGCTTAATCCACTGG
MLH1	GCCTTGGCACAGCATCAAACCA	ATGGCAAGGTCAAAGAGCGGTG
BRCA2	GGCTTCAAAAAGCACTCCAGATG	GGATTCTGTATCTCTTGACGTTCC
RAD51	TCTCTGGCAGTGATGTCCTGGA	TAAAGGGCGGTGGCACTGTCTA
Msh2	GAACAAAGGCGAGTATGAAGAGG	GCGTCTAAGTGAGCCAGCACAT
Mlh1	CCTCCAGGATGTATTTCACCCAG	ACGGACCATCTGGTAAGCGTAG
Brca2	GAGCACAGATGGAGACAGGGAA	CAGACGGAGATGTCGCCTTTCT
Rad51	TGGAGGCTGTTGCTTATGCACC	GCTGGTGAAACTCAGTTGCCGT
ACTB	GCTCCTCCTGAGCGCAAG	CATCTGCTGGAAGGTGGACA
Actb	TATGCTCTCCCTCACGCCATCC	GTCACGCACGATTTCCCTCTCAG
